# Structural Modifications at the C3 and C30 Positions of the Lupane Skeleton with Carbon-Centered Nucleophiles

**DOI:** 10.3390/molecules30153064

**Published:** 2025-07-22

**Authors:** Davide Castiglione, Gianfranco Fontana, Laura Castoldi, Vittorio Pace

**Affiliations:** 1 Department of Chemistry, University of Turin, Via P. Giuria 7, 10125 Turin, Italy; 2 Dipartimento di Scienze Biologiche, Chimiche e Farmaceutiche (STEBICEF), University of Palermo, Viale delle Scienze Ed. 17, 90128 Palermo, Italy; gianfranco.fontana@unipa.it; 3 Department of Pharmaceutical Sciences, General and Organic Chemisty Section “A. Marchesini”, University of Milan, Via Venezian 21, 20133 Milan, Italy; laura.castoldi@unimi.it; 4 Department of Pharmaceutical Sciences, Division of Pharmaceutical Chemistry, University of Vienna, Josef-Holaubek-Platz 2, 1090 Vienna, Austria

**Keywords:** lupeol, lupenone, natural substances, phytochemical, organolithium, organomagnesium, organoindium, carbenoid, homologation

## Abstract

Lupeol, a naturally occurring pentacyclic triterpenoid widely distributed in various medicinal plants, has attracted significant attention due to its diverse pharmacological properties. In this study, we report the synthesis and structural modification of 14 lupeol derivatives through selective functionalizations at C3 and C30 positions of the lupane skeleton, via the sequential chemoselective introduction of carbonyl moieties and the addition of organometallics. Emphasis has been given to the stereoselective alkylation at C3 using a range of carbanions, including organolithiums, organomagnesiums and organoindiums. The C30 position was modified through oxidative pathways to introduce several functionalities.

## 1. Introduction

**Scheme 1:**
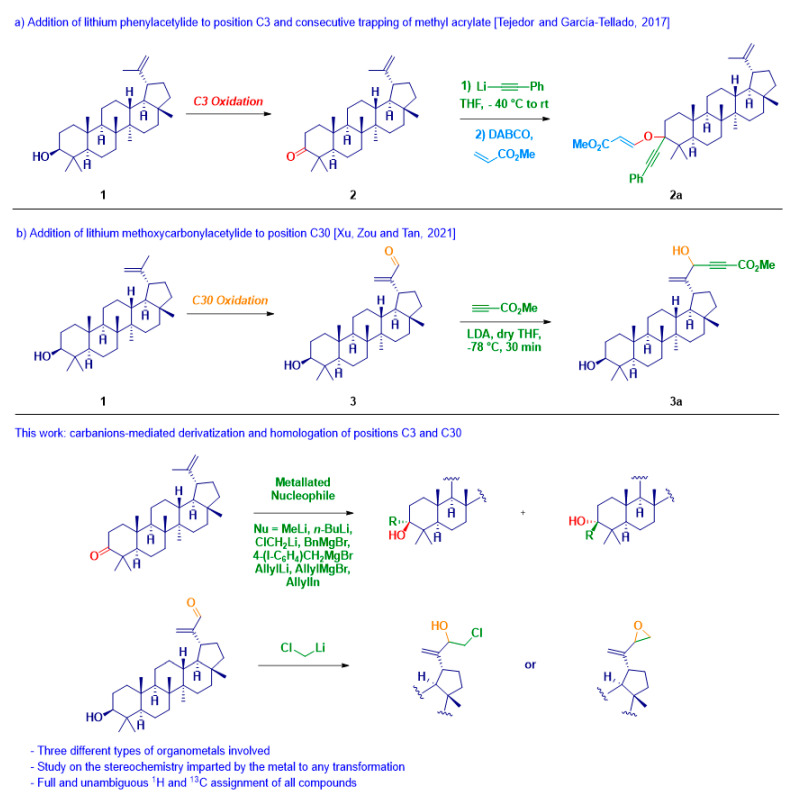
General context of the presented work [31,32].

Lupeol (**1**), also known as clerodol, fagarsterol or monoginol B, is a pentacyclic triterpene widely distributed in nature, which can be isolated from various plant species, including edible fruits and vegetables [1]. The peculiar biosynthetic process of lupeol is, to date, well-understood and represents one of the most complex natural biomolecular events [2]. Its lupane-type structure—derived from the cyclization of squalene oxide—consists of four six-membered rings and one five-membered ring, contains ten stereocentres and is characterized by two distinctive functional groups: a secondary hydroxyl group at position C3 and an olefin moiety at position C20. Lupeol is a natural compound of considerable interest in pharmacology [3]. Numerous in vivo and in vitro experiments have revealed its cardioprotective [4,5], antidiabetic [6,7], anti-inflammatory [8,9,10] and remarkable anticancer [11,12,13,14] properties. However, although it stands out as a promising lead compound for the development of experimental therapeutics, its pronounced lipophilicity and low degree of functionalization limit its clinical use and make structural modifications extensively needed to modulate its pharmaco-dynamic profile. Indeed, it is relevant to notice that many semisynthetic derivatives are more pharmacologically active than the parent terpene. For this purpose, several transformations have been performed on the lupane structure, including the preparation of esters [7,15,16,17,18] and (aza)ketones [19,20,21,22] at position C3, the synthesis of heterocyclic derivatives [23,24,25] and less common manipulations of the isopropenyl chain at position C19 [19,20,25,26]. In contrast, the installation of carbon chains at positions C3 and C30—convertible into electrophilic platforms via oxidation—through the addition of organometallic reagents is rare. However, this strategy has opened up pathways to congener homolupane triterpenoids (from betulin and betulinic acid) with intriguing antiproliferative activity against several tumor cell lines and selective TGR5 agonist activity [27,28,29]. As well, the C3 alkylated triterpenic acids are endowed with antitumor potential [19,30]. In fact, to the best of our knowledge, only two examples of organometallic-promoted functionalizations on the lupane skeleton have been reported to date. In 2017, Tejedor and Garcìa-Tellado performed the addition of lithium phenylacetylide to position C3 in lupenone (**2**) followed by a DABCO-promoted trapping of methyl acrylate en route to a benzoannulation of ring A (Scheme 1, path a) [31]. More recently, Xu, Zou and Tan reported the addition of lithium methoxycarbonylacetylide to the position C30—after a chemoselective oxidation of the allyl moiety into 30-formyl lupeol (**3**)—for the preparation of alcohol **3a** as a suitable substrate for their redox isomerization of propargylic alcohols (Scheme 1, path b) [32]. In light of this shortcoming, we report here a study on the preparation of (homologated) carbo-derivatives of lupeol, highlighting the effect dictated by the different nature of the organometallic nucleophiles involved and the stereochemistry of the products.

## 2. Results and Discussion

**Scheme 2:**
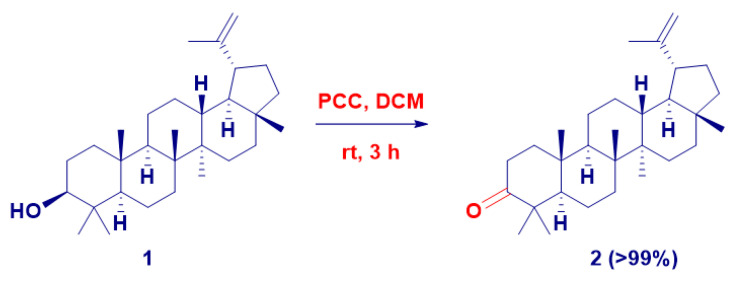
Preparation of lupenone via oxidation of position C3 with PCC.

Lupeol was isolated from the cyclohexane extract of commercial lupin seed husks, and its structure was confirmed by comparison with data reported in the literature [33]. Lupenone (**2**), a precursor of C3-alkylated products, was smoothly prepared via the oxidation of **1** with pyridinium chlorochromate (PCC) in DCM (Scheme 2) [19]. Operating under harsher conditions, e.g., Jones reagent, could be detrimental to the chemical integrity of ring E, which is sensitive to strongly acidic environments [34,35].

The synthesis of the new derivatives **4**–**15** starting from compounds **2** and **16** and of **17** from lupeol **1** are described below. The results obtained are summarized in Table 1. All the obtained compounds were fully characterized by spectroscopic methods, including 1D and 2D NMR spectroscopy that enabled the complete and unambiguous assignment of all the signals (see Supplementary Materials).

**Scheme 3:**
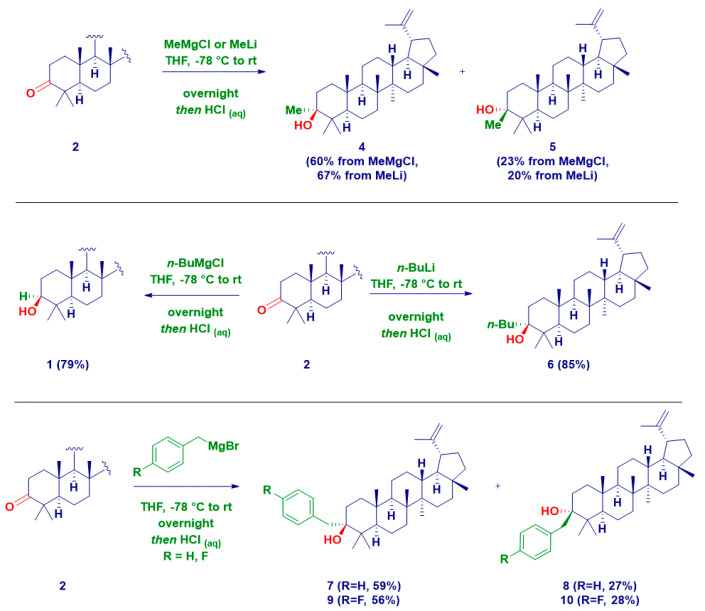
Installation of methyl, *n*-butyl and (4-fluoro)benzyl fragments at position C3.

The installation of a methyl chain through the reaction of **2** with MeMgCl smoothly afforded the two diastereomers 3-methyl-lupeol (**4**) and 3-methyl-3-epi-lupeol (**5**) with an overall yield of 83% (Scheme 3, Table 1 entries 3, 4). Notably, the 3-β-ol epimer was generated as a major product with 44% diastereomeric excess (**4**/**5** ratio = 72:28), showing a preferential nucleophile attack on the *Re* face of the carbonyl group. By conducting the methylation reaction with the more nucleophilic methyllithium under analogous conditions, an increase in yield to 87% was observed (Table 1, entries 1, 2); additionally, stereoselectivity towards **4** was more predominant (d.e. = 54%), presumably owing to the better coordinating properties of lithium—over magnesium cation—which allow a stronger interaction with the carbonyl oxygen and thus promote the nucleophilic attack along the pseudo-axial trajectory. The identification of the absolute stereochemistry at C3 was made based on the reported criterion of the C3 chemical shift [29,30], that is, a downfield shift for the β-OH configuration with respect to the α-OH, and by a new criterion that we report here for the first time. This is the relevant downfield shift of C5 (from ca. 53 ppm to ca. 51 ppm), associated with a slight upfield shift of H5, originated by the deformation of the σ C-H bond electron density sterically induced by the 1,3 diaxial interaction with the C3, R bond (in the β-OH isomer). This approach has been applied to all the C3-alkylated derivatives described hereafter.

The installation of the *n*-butyl chain was more challenging. The reaction with *n*-BuMgCl, indeed, did not generate butyl derivatives. Instead, the original secondary hydroxyl function was regenerated via a Cowan–Mosher-type reduction [36], presumably due to the high steric hindrance provided by the two geminal methyl groups at position C4 (Table 1, entry 6). Noteworthily, the hydride addition yielded the original lupeol as a single epimer (d.e. > 99%). The addition of *n*-BuLi (less prone to reductive phenomena) afforded 3-butyl-lupen-3-β-ol (**6**) with a >99% diastereomeric excess (Table 1, entry 5).

A similar stereoselectivity was observed in the addition of benzyl- and 4-fluorobenzylmagnesium bromide (Scheme 3, below; Table 1, entries 7–10). In both cases, the corresponding carbinols derivatives were smoothly obtained. After reacting **2** with BnMgBr, 3-benzyl lupeol (**7**) and 3-benzyl-3-epi-lupeol (**8**) were isolated in 86% yield and 38% d.e., and the reaction with the fluorinated congener furnished 3-(4-fluorobenzyl)lupeol (**9**) and 3-(4-fluorobenzyl)-3-epi-lupeol (**10**) in comparable yield (84%) and 34% d.e. Both vinyl chloride and bromide failed to react with ketone **2**, probably due to the intrinsically lesser reactivity of C-sp2 carbon nucleophiles. Of note, the stereoselectivity of the Grignard reagent addition was not related with the steric hindrance of the approaching nucleophiles.

**Scheme 4:**
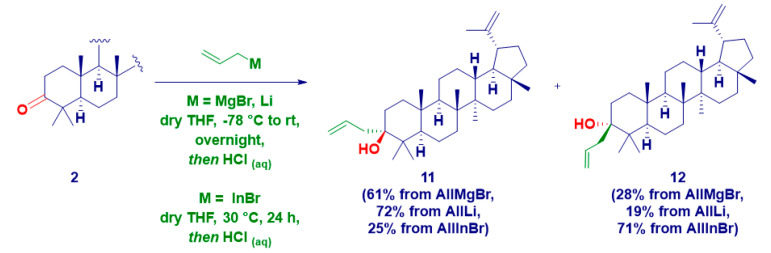
γ-allylation of lupenone.

In fact, by reacting lupenone with allylmagnesium bromide, 3-allyl-lupeol (**11**) and 3-allyl-3-*epi*-lupeol (**12**) were isolated in 89% overall yield and 36% d.e., whereas the addition of allyllithium—prepared in situ by Li-Sn exchange from allyltributylstannane and *n*-BuLi—yielded **11** and **12** in slightly higher yield (91%) but with 58% d.e. in favor of the same epimer (Scheme 4). Surprisingly, when the same transformation was performed by employing an organoindium reagent, the diastereoselectivity was reversed. The addition of allylindium bromide, prepared in Barbier conditions from allyl bromide and indium metal in THF, generated the 3-α homoallyl alcohol **12** as the major product, with 48% d.e. (96% overall yield).

**Scheme 5:**
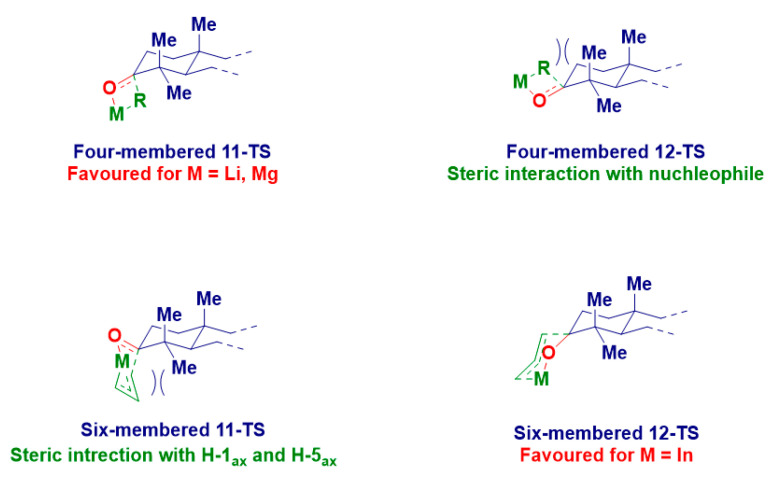
Possible transition states involved in γ-allylation of lupenone.

This kind of stereoselectivity is in agreement with the one observed for the allylation reaction of a set of 4-substituted cyclohexanones [36]. On the other hand, the stereoselectivity observed with magnesium is similar to the one reported for the allylation of 3-oxo-oleanolic acid [19,30], 3-oxo-ursolic acid [30] and 3-oxo-betulinic acid [28]. Notably, while our results align with those observed with the addition of allylmagnesium bromide to other triterpene ketones and of allylindium to conformationally biased cyclohexanones, they diverge with regard to the addition of methyl, allyl and butyl lithium. These organometallic reagents showed a definite preference for the equatorial attack due to a steric control approach [37]. However, in our case, as well as in the other allylation reactions reported [19,28,30], the presence of an axial methyl group at C4 position may originate a stronger steric tension toward the incoming reagent with respect to the C1 and C5 axial hydrogens. This effect is probably more pronounced in the case of the more Lewis-acidic organolithium and organomagnesium reagents with respect to the corresponding organoindium species. In the first two cases, an early more compact four-membered transition state could be postulated, while, in the case of indium, a looser six-membered transition state would be less sensitive to the presence of the axial methyl, instead evolving through a normal steric-controlled approach (equatorial attack preferred). The fact that the stereoselectivity observed for allyl-MgBr lies in between allyl-Li on one side and allyl-In on the other is coherent with the multiple mechanism of interaction of Grignard reagents [38] with a carbonyl group, i.e., four-membered bimolecular TS, six-membered trimolecular TS without allylic rearrangement and, finally, bimolecular six-membered cyclic TS with allylic rearrangement (Scheme 5). This hypothesis is supported by the observation that the stereoselectivity obtained with the alkylmagnesium reagents is only slightly affected by the size of the alkyl group, ranging from methyl to 4-fluorobenzyl, and by its higher value obtained with lithium with respect to magnesium, the former being more Lewis acidic than the latter. Further, the intervention of different association and clustering phenomena for the organometallic reagents involved, particularly for Mg^38^ and In species in THF [39], cannot be ruled out a priori.

**Scheme 6:**
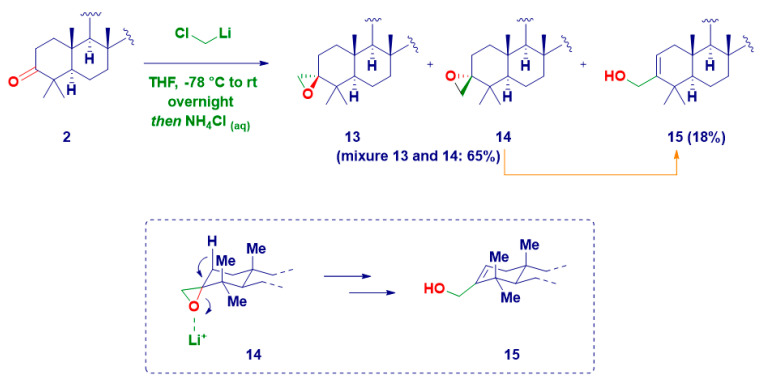
Carbenoid-mediated one-carbon homologation of lupenone.

To further implement the functionalities installed at position C3 of the lupane structure, we also accomplished the preparation of one-carbon homologated C3 spiro-epoxy derivatives. The initial tentative epoxidation via a Corey–Chaykovsky-type reaction [40,41] with trimethylsulphonium iodide did not afford the intended products, although diverse bases (NaH, LDA, potassium *t*-Pentoxide) and conditions were tested. Arguably, due to the steric hindrance of both ylide and substrate, no reaction occurred and the unreacted ketone was entirely recovered. Nonetheless, the addition of the less hindered chloromethyllithium—generated in Barbier conditions from MeI and MeLi-LiBr [42,43]—was successful (Scheme 6). As previously reported, the addition of versatile halomethyllithium carbenoids to carbonyl scaffolds provides an excellent synthetic strategy for the one-pot preparation of homologated epoxides [44,45,46,47,48]. Again, nucleophilic attack was predominant on the *Re*-face of the ketone, and, through the ring-closure of the two β-chloro alkoxide intermediates, spiro-epoxides **13** and **14** were generated and thus isolated as a mixture of chromatographically inseparable epimers. Unexpectedly, the majority of the strained spiro system of **14** spontaneously converted into the allyl alcohol **15** via the deprotonation of the axial hydrogen in position C2—anti-periplanar to the oxygen atom—and consequential ring opening, presumably favored by a lithium–oxygen coordination. It is worth emphasizing that the reaction proceeded cleanly and with total chemoselectivity despite the presence of the vinyl functionality, known to potentially undergo Simmons–Smith-type cyclopropanations with carbenoids [49,50].

**Scheme 7:**
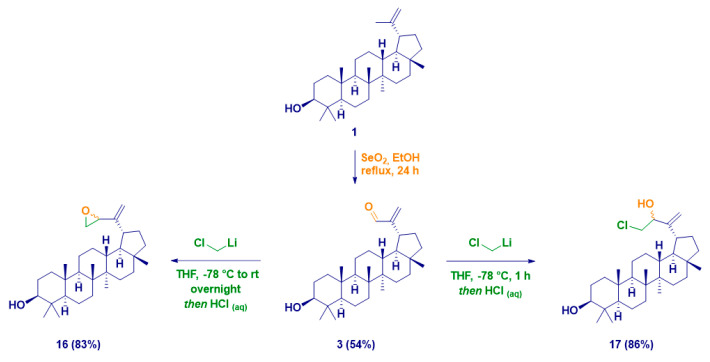
Oxidation and chemoselective carbenoid-mediated homologation of position C30.

Motivated by the interesting results obtained from the manipulation of position C3, we aimed for the intricate functionalization of the C30 methyl group. Taking advantage of the unique reactivity inherent in the allylic moiety, we could easily prepare α, β-unsaturated aldehyde **3** by the direct oxidation of lupeol with SeO_2_ in EtOH, thus introducing a new electrophilic platform on the lupane scaffold. A second chloromethyllithium-mediated epoxidation yielded epimeric epoxides **16**, which were unfortunately inseparable, in 83% yield and with a 1:1.7 ratio (calculated from the ^1^H-NMR spectrum of the purified mixture) (Scheme 7). Nevertheless, complete and unambiguous ^1^H and ^13^C chemical shift assignments were successfully achieved. Ultimately, to further exploit the synthetic possibilities offered by the lithium carbenoid, the preparation of (homologated) chlorohydrins was also tested. By conducting the addition of chloromethyllithium at −78 °C and upon acidic quenching at low temperature within one hour, the cyclization of the intermediate lithium alkoxide was prevented and, as desired, the two halohydrins **17** were generated in comparable yield (86%) and diastereomeric ratio, ca. 1:1.7 (calculated from the ^1^H-NMR spectrum of the crude mixture). Once again, the separation of the two diastereoisomers proved particularly challenging, but the isolation of a portion of one of the epimers enabled the characterization of this novel halo-homolupane terpenoid. Some additional aspects merit mention: (a) Protection of the hydroxyl group at position C3 was unnecessary; instead, an extra equivalent of carbanion was found to be an effective alternative approach. At the end of the operation, the chemical integrity of the secondary alcohol was fully preserved; (b) In both transformations, no trace of 1,4-addition products was observed, confirming the already-proven chemoselectivity of lithium halomethyl carbenoids [51,52,53]. In addition, the vinyl chain did not undergo concurrent cyclopropanation; (c) The determination of the absolute configuration of the new chiral centre at C30 by NMR analysis was somewhat arduous due to the lack of significant evidence obtainable from NOESY spectra. For this reason, a specific ab initio computational protocol is being developed and will be illustrated in detail in a separate article.

## 3. Materials and Methods

^1^H- and ^13^C-NMR spectra were recorded on a Jeol (Peabody, MA, USA) ECZR600 (600 MHz for ^1^H and 150 MHz for ^13^C) spectrometer and a Bruker (Billerica, MA, USA) Avance Neo 400 spectrometer (400 MHz for ^1^H and 100 MHz for ^13^C). The center of the (residual) solvent signal was used as an internal standard, which was related to TMS with δ 7.26 ppm (^1^H-NMR in CDCl_3_) and δ 77.0 ppm (^13^C-NMR in CDCl_3_). Spin–spin coupling constants (*J*) are given in Hz. In all cases, the full and unambiguous assignment of all resonances was performed by the combined application of standard NMR techniques, such as APT, HSQC, HMBC, COSY and NOESY experiments. Melting points were determined on a Reichert–Kofler (Vienna, Austria) hot-stage microscope. Mass spectra were obtained on a Bruker maXis 4G instrument (ESI-TOF, HRMS). All reactions were performed under an inert atmosphere of argon using standard Schlenk techniques. THF was distilled over Na/benzophenone. Chemicals were purchased from Sigma Aldrich (Saint Louis, MO, USA), Acros (Verona, Italy), Alfa Aesar (Heysham, UK), Fluorochem (Hadfield, UK) and TCI Europe (Zwijndrecht, Belgium). Solutions were evaporated under reduced pressure with a rotary evaporator. For column chromatography, silica Gel 60 (0.04–0.063 mm) was used. TLC was carried out on aluminum sheets precoated with silica gel 60F254 (Merchery-Nagel (Düren, Germany), Merk); the spots were visualized under UV light (λ = 254 nm) and/or KMnO_4 (aq.)_ was used as revealing system. Synthetic procedures, experimental and NMR data and copies of spectra are available in the Supporting Information.

**Extraction of lupeol (1).** First, 1000 g of lupin seed husks was washed with water, air-dried for 48 h and then ground to obtain 360 mg of dry granulate. This was suspended in cyclohexane (500 mL per 200 mg of granulate), stirred at 60 °C (by oil bath) for 24 h and filtered, and the resulting solution was finally evaporated under reduced pressure to obtain a straw-yellow solid. The granulate was subjected to the same process twice more and the extracts combined. Lupeol (1.34 g) was isolated from the dry extract (2.20 g) as a white solid after chromatography on silica gel (90:10 *v*/*v*, *n*-hexane/ethyl acetate). Melting point and NMR data correspond to those reported in the literature [33,34,35].

**Lupenone (2).** Under argon atmosphere, PCC (1015.0 mg, 4.70 mmol, 2 equiv) was added to a solution of lupeol (1000.0 mg, 2.34 mmol, 1 equiv) in dry DCM (40 mL) at room temperature and the mixture was stirred for 3 h, then concentred under reduced pressure and filtered through celite. The final solution was evaporated to afford compound **2** quantitatively (986 mg, >99%) as a white solid. Melting point and NMR data correspond to those reported in the literature [24].

**3-methyl lupeol (4) and 3-epi-3-methyl lupeol (5)**. Under argon atmosphere, MeMgCl (0.23 mL of a solution 3.0 M in THF, 0.7 mmol, 2.0 equiv) or MeLi (0.43 mL of a solution 1.6 M in diethyl ether, 0.7 mmol, 2.0 equiv) was added dropwise to a solution of lupenone (150 mg, 0.35 mmol, 1.0 equiv) in dry THF (5 mL) at −78 °C, and the resulting mixture was allowed to slowly warm to room temperature and stirred overnight, before being quenched with 1M HCl (3 mL). The resulting mixture was extracted 3 times with Et_2_O; the organic phases were combined, washed with brine, dried over anhydrous Na_2_SO_4_ and concentrated in vacuo.

From MeMgCl, compound **4** was obtained in 60% yield (92.11 mg) as a white solid (m.p.: 165–166 °C) and compound **5** was obtained in 23% yield (35.7 mg) as a white solid (m.p.: 132–133 °C) after chromatography on silica gel (92:8 *v*/*v*, *n*-hexane/ethyl acetate). Overall yield: 83%, d.e.: 44%.

From MeLi, compound **4** was obtained in 67% yield (103.3 mg) as a white solid (m.p.: 165–166 °C) and compound **5** was obtained in 20% yield (30.9 mg) as a white solid (m.p.: 132–133 °C) after chromatography on silica gel (92:8 *v*/*v*, *n*-hexane/ethyl acetate). Overall yield: 87%, d.e.: 54%.

HRMS (ESI) compound **4**, *m*/*z*: calc. for C_31_H_52_OH+: 4,414,091 [M + H]+; found: 4,414,095. HRMS (ESI) compound **5**, *m*/*z*: calc. for C_31_H_52_OH+: 4,414,091 [M + H]+; found: 4,414,093.

**Cowan–Mosher-type reduction of lupenone with *n*-BuMgCl.** Under argon atmosphere, *n*-BuMgCl (0.35 mL of a solution 2.0 M in THF, 0.7 mmol, 2.0 equiv) was added dropwise to a solution of lupenone (150 mg, 0.35 mmol, 1.0 equiv) in dry THF (5 mL) at −78 °C, and the resulting mixture was allowed to slowly warm to room temperature and stirred overnight, before being quenched with 1M HCl (3 mL). The resulting mixture was extracted 3 times with Et_2_O; the organic phases were combined, washed with brine, dried over anhydrous Na_2_SO_4_ and concentrated in vacuo. Compound 1 was obtained in 79% yield (118.0 mg) as a white solid after chromatography on silica gel (90:10 *v*/*v*, *n*-hexane/ethyl acetate).

**3-butyl lupeol (6)**. Under argon atmosphere, *n*-BuLi (0.28 mL of a solution 2.5 M in hexane, 0.7 mmol, 2.0 equiv) was added dropwise to a solution of lupenone (150 mg, 0.35 mmol, 1.0 equiv) in dry THF (5 mL) at −78 °C, and the resulting mixture was allowed to slowly warm to room temperature and stirred overnight, before being quenched with 1M HCl (3 mL). The resulting mixture was extracted 3 times with Et_2_O; the organic phases were combined, washed with brine, dried over anhydrous Na_2_SO_4_ and concentrated in vacuo. Compound **6** was obtained in 85% yield (143.6 mg) as a white solid (m.p.: 157–158 °C) after chromatography on silica gel (95:5 *v*/*v*, *n*-hexane/ethyl acetate). HRMS (ESI), *m*/*z*: calc. for C_34_H_58_OH+: 4,834,560 [M + H]^+^; found: 4,834,563.

**3-benzyl lupeol (7) and 3-epi-3-benzyl lupeol (8).** Under argon atmosphere, benzylmagnesium chloride (0.7 mL of a solution 1.0 M in diethyl ether, 0.7 mmol, 2.0 equiv) was added dropwise to a solution of lupenone (150 mg, 0.35 mmol, 1.0 equiv) in dry THF (5 mL) at −78 °C, and the resulting mixture was allowed to slowly warm to room temperature and stirred overnight, before being quenched with 1M HCl (3 mL). The resulting mixture was extracted 3 times with Et_2_O; the organic phases were combined, washed with brine, dried over anhydrous Na_2_SO_4_ and concentrated in vacuo. Compound **7** was obtained in 59% yield (107.3 mg) as a white solid (m.p.: 181–182 °C) and compound **8** was obtained in 27% yield (48.2 mg) as a white solid (m.p.: 169–170 °C) after chromatography on silica gel (96:4 *v*/*v*, *n*-hexane/ethyl acetate). Overall yield: 86%, d.e.: 38%.

HRMS (ESI) compound **7**, *m*/*z*: calc. for C_37_H_56_OH^+^: 517,4404 [M + H]^+^; found: 5,174,401.

HRMS (ESI) compound **8**, *m*/*z*: calc. for C_37_H_56_OH^+^: 517,4404 [M + H]^+^; found: 5,174,402.

**3-(4-fluorobenzyl)lupeol (9) and 3-epi-3-(4-fluorobenzyl)lupeol (10).** Under argon atmosphere, the solution of freshly prepared 4-fluorobenzylmagnesium bromide was added dropwise to a solution of lupenone (150 mg, 0.35 mmol, 1.0 equiv) in dry THF (5 mL) at −78 °C, and the resulting mixture was allowed to slowly warm to room temperature and stirred overnight, before being quenched with 1M HCl (3 mL). The resulting mixture was extracted 3 times with Et_2_O; the organic phases were combined, washed with brine, dried over anhydrous Na_2_SO_4_ and concentrated in vacuo. Compound **9** was obtained in 56% yield (105.3 mg) as a white solid (m.p.: 188–189 °C) and compound **10** was obtained in 28% yield (51.9 mg) as a white solid (m.p.: 173–174 °C) after chromatography on silica gel (96:4 *v*/*v*, *n*-hexane/ethyl acetate). Overall yield: 84%, d.e.: 34%.

HRMS (ESI) compound **9**, *m*/*z*: calc. for C_33_H_54_FOH^+^: 467,4247 [M + H]^+^; found: 4,674,244. HRMS (ESI) compound **10**, *m*/*z*: calc. for C_33_H_54_FOH^+^: 467,4247 [M + H]^+^; found: 4,674,242.

**Preparation of 4-fluorobenzylmagnesium bromide**. Under argon atmosphere, to a suspension of powdered magnesium metal (35.7 mg, 1.46 mmol, 4.2 equiv) in 4 mL of anhydrous diethyl ether, a drop of 1.2 dibromoethane and a grain of I_2_ were added, and the red mixture was vigorously stirred until a straw-yellow color appeared. 4-fluorobenzyl bromide (0.09 mL, 139.9 mg, 0.74 mmol, 2.1 equiv) in dry diethyl ether (2.0 mL) was slowly added over 15 min, and the final mixture was refluxed for 3 h. Upon completion, the solution was cooled to 0 °C by ice-water bath and collected by syringe under a constant flow of argon.

**3-allyl lupeol (11) and 3-epi-3-allyl lupeol (12).**

From allylmagnesium bromide: Under argon atmosphere, allylmagnesium bromide (0.7 mL of a solution 1.0 M in diethyl ether, 0.7 mmol, 2.0 equiv) was added dropwise to a solution of lupenone (150 mg, 0.35 mmol, 1.0 equiv) in dry THF (5 mL) at −78 °C, and the resulting mixture was allowed to slowly warm to room temperature and stirred overnight, before being quenched with 1M HCl (3 mL). The resulting mixture was extracted 3 times with Et_2_O; the organic phases were combined, washed with brine, dried over anhydrous Na_2_SO_4_ and concentrated in vacuo. Compound **11** was obtained in 61% yield (98.9 mg) as a white solid (m.p.: 137–138 °C) and compound **12** was obtained in 28% yield (46.5 mg) as a white solid (m.p.: 149–150 °C) after chromatography on silica gel (95:5 *v*/*v*, *n*-hexane/ethyl acetate). Overall yield: 89%, d.e.: 36%.

From allyllithium: Following a previously reported procedure, under argon atmosphere, *n*-BuLi (0.28 mL of a solution 2.5 M in hexane, 0.7 mmol, 2.0 equiv) was added dropwise to a solution of allyltributylstannane (0.23 mL, 245.0 mg, 0.74 mmol, 2.1 equiv) in dry THF (3 mL) at −78 °C, and the resulting mixture was stirred for a further 1 h. The solution of freshly prepared allyllithium was added dropwise to a solution of lupenone (150 mg, 0.35 mmol, 1.0 equiv) in dry THF (5 mL) at −78 °C, and the resulting mixture was allowed to slowly warm to room temperature and stirred overnight, before being quenched with 1M HCl (3 mL). The resulting mixture was extracted 3 times with Et_2_O; the organic phases were combined, washed with brine, dried over anhydrous Na_2_SO_4_ and concentrated in vacuo. Compound **11** was obtained in 72% yield (117.5 mg) as a white solid (m.p.: 149–150 °C) and compound **12** was obtained in 19% yield (31.2 mg) as a white solid (m.p.: 137–138 °C) after chromatography on silica gel (95:5 *v*/*v*, *n*-hexane/ethyl acetate). Overall yield: 91%, d.e.: 58%.

From allylindium bromide: Allylbromide (0.47 mL, 652.1 mg, 5.39 mmol, 15.4 equiv) was added dropwise to a solution of lupenone (150 mg, 0.35 mmol, 1.0 equiv) and powdered metal indium (618.9 mg, 5.39 mmol, 15.4 equiv) in dry THF (7.5 mL) at room temperature, and the solution was stirred for 24 h at 30 °C. The final mixture was then cooled to 0 °C by ice bath and quenched with 1M HCl (3 mL). The residual metal indium was filtered off and the resulting mixture was extracted 3 times with Et_2_O; the organic phases were combined, washed with brine, dried over anhydrous Na_2_SO_4_ and concentrated in vacuo. Compound **11** was obtained in 25% yield (40.8 mg) as a white solid (m.p.: 149–150 °C) and compound **12** was obtained in 71% yield (116.1 mg) as a white solid (m.p.: 137–138 °C) after chromatography on silica gel (95:5 *v*/*v*, *n*-hexane/ethyl acetate). Overall yield: 96%, d.e.: 48%.

HRMS (ESI) compound **11**, *m*/*z*: calc. for C_37_H_55_OH^+^: 5,354,310 [M + H]^+^; found: 5,354,308. HRMS (ESI) compound **12**, *m*/*z*: calc. for C_37_H_55_OH^+^: 5,354,310 [M + H]^+^; found: 5,354,313.

**3-(R)-3,1′-epoxy-lup-20-ene (13), 3-(S)-3,1′-epoxy-lup-20-ene (14) and 3-hydroxymethyl-lup-1,20-diene (15)**. Under argon atmosphere, MeLi-LiBr (0.44 mL of a solution 2.2 M in diethyl ether, 0.98 mmol, 2.8 equiv) was added dropwise to a solution of lupenone (150 mg, 0.35 mmol, 1.0 equiv) and chloroiodomethane (0.08 mL, 185.2 mg, 1.05 mmol, 3.0 equiv) in dry THF (5 mL) at −78 °C, and the resulting mixture was allowed to slowly warm to room temperature and stirred overnight, before being quenched with 1M HCl (3 mL). The resulting mixture was extracted 3 times with Et_2_O; the organic phases were combined, washed with brine, dried over anhydrous Na_2_SO_4_ and concentrated in vacuo. The mixture of compounds **13** and **14** was obtained in 65% yield (99.8 mg) as a white solid (m.p.: range 165–174 °C) (ratio **13**/**14** calculated from ^1^H-NMR: 1:0.17) and compound **15** was obtained in 18% yield (27.6 mg) as a white solid (m.p.: 248 °C) after chromatography on silica gel (gradient from 95:5 to 90:10 *v*/*v*, *n*-hexane/ethyl acetate). Overall yield: 83%.

HRMS (ESI) compounds **13** and **14**, *m*/*z*: calc. for C_31_H_50_OH^+^: 4,393,934 [M + H]^+^; found: 4,393,939. HRMS (ESI) compound **15**, *m*/*z*: calc. for C_31_H_50_OH^+^: 4,393,934 [M + H]^+^; found: 4,393,932.

**30-formyl lupeol (3).** A solution of lupeol (500 mg, 1.17 mmol, 1.0 equiv) and SeO_2_ (194.2 mg, 1.75 mmol, 1.5 equiv) in dry ethanol (20 mL) was refluxed for 48 h. The resulting mixture was filtered through celite and extracted 3 times with Et_2_O; the organic phases were combined, washed with brine, dried over anhydrous Na_2_SO_4_ and concentrated in vacuo. Compound **3** was obtained in 54% yield (278.43 mg) as a white solid after chromatography on silica gel (85:15 *v*/*v*, *n*-hexane/ethyl acetate). Melting point and NMR data correspond to those reported in the literature [25,33].

**1′,30-epoxy-lupeol (16).** Under argon atmosphere, MeLi-LiBr (0.34 mL of a solution 2.2 M in diethyl ether, 0.76 mmol, 2.8 equiv) was added dropwise to a solution of 30-formyl-lupeol (**15**) (120 mg, 0.27 mmol, 1.0 equiv) and chloroiodomethane (0.06 mL, 142.9 mg, 0.81 mmol, 3.0 equiv) in dry THF (5 mL) at −78 °C, and the resulting mixture was allowed to slowly warm to room temperature and stirred overnight, before being quenched with 1M HCl (3 mL). The resulting mixture was extracted 3 times with Et_2_O; the organic phases were combined, washed with brine, dried over anhydrous Na_2_SO_4_ and concentrated in vacuo. Compound **16** was obtained in 83% yield (101.9 mg) as a white solid (m.p.: 198–212 °C) (ratio calculated from ^1^H-NMR 1:0.6) after chromatography on silica gel (90:10 *v*/*v*, *n*-hexane/ethyl acetate). HRMS (ESI), *m*/*z*: calc. for C_31_H_50_O_2_H^+^: 4,553,884 [M + H]^+^; found: 4,553,888.

**1′-chloro-30-hydroxy-lupeol (17).** Under argon atmosphere, MeLi-LiBr (0.34 mL of a solution 2.2 M in diethyl ether, 0.76 mmol, 2.8 equiv) was added dropwise to a solution of 30-formyl-lupeol (**15**) (120 mg, 0.27 mmol, 1.0 equiv) and chloroiodomethane (0.06 mL, 142.9 mg, 0.81 mmol, 3.0 equiv) in dry THF (5 mL) at −78 °C, and the resulting mixture was stirred at low temperature for 1 h, before being quenched with 1M HCl (3 mL). The resulting mixture was extracted 3 times with Et_2_O; the organic phases were combined, washed with brine, dried over anhydrous Na_2_SO_4_ and concentrated in vacuo. Compound **17** was obtained in 86% yield (114.1 mg) as a white solid (m.p.: 241–253 °C) (ratio calculated from ^1^H-NMR spectrum of the crude reaction mixture: 1:0.6) after chromatography on silica gel (85:15 *v*/*v*, *n*-hexane/ethyl acetate). HRMS (ESI), *m*/*z*: calc. for C_31_H_51_ClO_2_H^+^: 4,913,650 [M + H]^+^; found: 4,913,647.

## 4. Conclusions

Fourteen new derivatives of the triterpene phytochemical lupeol have been synthetized and characterized. They have been obtained by modification at the C3 and C30 positions. This set of compounds may also have biological relevance, as recently demonstrated for a number of C3 alkyl derivatives of betulinic acid [37] endowed with significant binding capacity toward the TGR5 receptor and for C3 allyl oleanolic acid [30] and ursolic acid [19], showing interesting antitumor activities toward hepatocarcinoma and leukemia cell lines. Both the epimers at C3 have been isolated with a prevalence of the alpha hydroxyl stereoisomer with organolithium and, in a lesser extent, with organomagnesium. The stereochemistry of the C3 allyl-substituted derivative can be controlled by the choice of the allyl-metal species: lithium giving rise to the axial attack and indium, on the contrary, to the equatorial attack. This kind of stereoselectivity was interpreted by invoking two different steric approaches, one operating via a four-membered TS (for R-Li and RMgBr) and the other operating via a six-membered TS (allyl-In and, in part, allyl-MgBr). The presence of an axial-oriented methyl fragment on C4 of the A ring of the triterpene disfavors the β-equatorial approach of the organometallic, rather favoring the α-axial one.

## Figures and Tables

**Table 1:** Addition reaction of diverse organometallic species (RM) to lupenone **2**.

Entry	Product	R	M	Temp.	Yield%	d.e.%
1	**4**	Me	Li	−78 °C to rt	67	47
2	**5**				20	
3	**4**		MgCl		60	37
4	**5**				23	
5	**6**	n-Bu	Li		85	>99
6	**1**		MgX		79	>99
7	**7**	benzyl			59	32
8	**8**				27	
9	**9**	4-F-benzyl			56	28
10	**10**				28	
11	**11**	allyl	Li		72	53
12	**12**				19	
13	**11**		MgBr		61	33
14	**12**				28	
15	**11**		In	rt	25	46
16	**12**				71	

## Data Availability

The data supporting this article have been included as part of the Supplementary Information.

## Supplementary Materials

The following supporting information can be downloaded at: https://www.mdpi.com/article/10.3390/molecules30153064/s1, Instrumentation and general analytical methods, extraction of lupeol, preparation, purification and HRMS data of all compounds, full ^1^H- and ^13^C-NMR assignment for all the products, copies of ^1^H- and ^13^C-NMR spectra for all products. Refs. [24,25,33,34,54] are cited in the Supplementary Materials.

## Abbreviations

The following abbreviations are used in this manuscript:

NMR	Nuclear magnetic resonance
HRMS	High-resolution mass spectrometry
THF	Tetrahydrofurane
DABCO	1,4-diazabicyclo[2.2.2]octane
LDA	Lithium diisopropylamide
PCC	Pyridium chlorochromate
DCM	Dichloromethane
TS	Transition state
TGR5	Takeda G protein-coupled receptor 5
