# Supplementary material for: Structural Modifications at the C3 and C30 Positions of the Lupane Skeleton with Carbon-Centered Nucleophiles
Source: Molecules. 2025 Jul 22;30(15):3064. doi: 10.3390/molecules30153064 (PMC12348400; doi:10.3390/molecules30153064)
Supplement: Supplementary file 1 [file molecules-30-03064-s001.zip › molecules-3739651-supplementary.pdf]

Supporting information

Structural Modifications at the C3 and C30 Positions of the Lupane Skeleton with Carbon-Centered Nucleophiles

Davide Castiglione [1,*], Gianfranco Fontana [2], Laura Castoldi [3] and Vittorio Pace [1,4,*]

1 Department of Chemistry, University of Turin, Via P. Giuria 7, 10125 Turin, Italy
2 Dipartimento di Scienze Biologiche, Chimiche e Farmaceutiche (STEBICEF), University of Palermo, Viale delle Scienze Ed. 17, 90128 Palermo, Italy; gianfranco.fontana@unipa.it
3 Department of Pharmaceutical Sciences, General and Organic Chemisty Section "A. Marchesini", University of Milan, Via Venezian 21, 20133 Milan, Italy; laura.castoldi@unimi.it
4 Department of Pharmaceutical Sciences, Division of Pharmaceutical Chemistry, University of Vienna, Josef-Holaubek-Platz 2, 1090 Vienna, Austria
* Correspondence: davide.castiglione@unito.it (D.C.); vittorio.pace@univie.ac.at (V.P.); Tel.: +39-011-6707934 (V.P.)

Instrumentation and General Analytical Methods 2
Extraction of lupeol (1) 3
Preparation, purification and HRMS data of all compounds 4
Full ^{1}H- and ^{13}C-NMR assignment for all the products 10
Copies of ^{1}H- and ^{13}C-NMR Spectra for all the products 21
References 36

Instrumentation and General Analytical Methods

^{1}H- and ^{13}C-NMR spectra were recorded on a Jeol ECZR600 (600 MHz for ^{1}H and 150 MHz for ^{13}C) spectrometer and a Bruker Avance Neo 400 spectrometer (400 MHz for ^{1}H and 100 MHz for ^{13}C). The center of the (residual) solvent signal was used as an internal standard which was related to TMS with δ 7.26 ppm (^{1}H-NMR in $CDCl_3$) and δ 77.0 ppm (^{13}C-NMR in $CDCl_3$). Spin-spin coupling constants (*J*) are given in Hz. In all cases, full and unambiguous assignment of all resonances was performed by combined application of standard NMR techniques, such as APT, HSQC, HMBC, COSY and NOESY experiments. Melting points were determined on a Reichert–Kofler hot-stage microscope. Mass spectra were obtained on a Bruker maXis 4G instrument (ESI-TOF, HRMS). All reactions were performed under an inert atmosphere of argon using standard schlenk techniques. THF was distilled over Na/benzophenone. Chemicals were purchased from SigmaAldrich, Acros, Alfa Aesar, Fluorochem and TCI Europe. Solutions were evaporated under reduced pressure with a rotary evaporator. For column chromatography, silica Gel 60 (0.04-0.063 mm) was used. TLC was carried out on aluminium sheets precoated with silica gel 60F254 (Merchery-Nagel, Merk); the spots were visualized under UV light (λ = 254 nm) and/or $KMnO_{4\ (aq.)}$ was used as revealing system.

Extraction of lupeol (1)

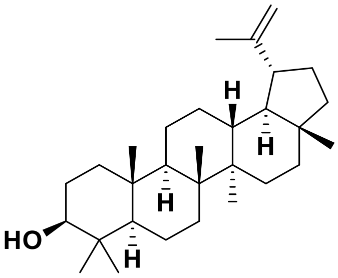

1000 g of lupin seed husks were washed with water, air-dried for 48 hours and then grinded to obtain 360 mg of dry granulate. This was suspended in cyclohexane (500 mL per 200 mg of granulate), stirred at 60°C (by oil bath) for 24 hours, filtered and the resulting solution was finally evaporated under reduced pressure to obtain a straw-yellow solid. The granulate was subjected to the same process twice more and the extracts combined. Lupeol (1.34 g) was isolated from the dry extract (2.20 g) as white solid after chromatography on silica gel on (90:10 v/v, n-hexane/ethyl acetate). Melting point and NMR data correspond to those reported in the literature.[1]

Preparation, purification and HRMS data of all compounds

Lupenone (2)

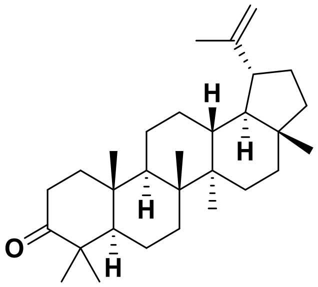

Under argon atmosphere, PCC (1015.0 mg, 4.70 mmol, 2 equiv) was added to a solution of lupeol (1000.0 mg, 2.34 mmol, 1 equiv) in dry DCM (40 mL) at room temperature and the mixture was stirred for 3 hours, then concentred under reduced pressure and filtered through celite. The final solution was evaporated to afford compound **2** quantitatively (986 mg, >99%) as white solid. Melting point and NMR data correspond to those reported in the literature.[2]

3-methyl lupeol (4) and 3-epi-3-methyl lupeol (5)

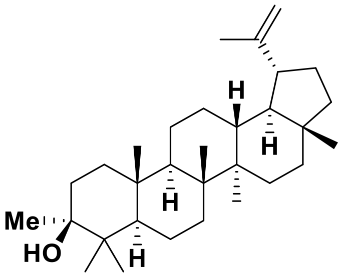

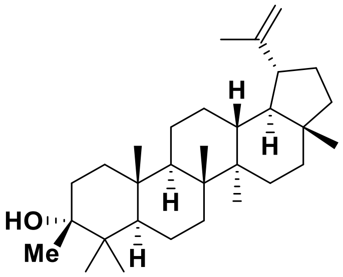

Under argon atmosphere, MeMgCl (0.23 mL of a solution 3.0 M in THF, 0.7 mmol, 2.0 equiv) or MeLi (0.43 mL of a solution 1.6 M in diethyl ether, 0.7 mmol, 2.0 equiv) was added dropwise to a solution of lupenone (150 mg, 0.35 mmol, 1.0 equiv) in dry THF (5 mL) at −78 °C and the resulting mixture was allowed to slowly warm to room temperature and stirred overnight, before being quenched with 1M HCl (3 mL). The resulting mixture was extracted 3 times with Et_2O, the organic phases were combined, washed with brine, dried over anhydrous Na_2SO_4 and concentrated in vacuo.

From MeMgCl: compound **4** was obtained in 60% yield (92.11 mg) as white solid (m.p.: 165-166 °C) and compound **5** was obtained in 23% yield (35.7 mg) as white solid (m.p.: 132-133 °C) after chromatography on silica gel (92:8 v/v, *n*-hexane/ethyl acetate). Overall yield: 83%, *d.e.*: 44%.

From MeLi: compound 4 was obtained in 67% yield (103.3 mg) as white solid (m.p.: 165-166 °C) and compound **5** was obtained in 20% yield (30.9 mg) as white solid (m.p.: 132-133 °C) after chromatography on silica gel (92:8 v/v, *n*-hexane/ethyl acetate). Overall yield: 87%, *d.e.*: 54%.

HRMS (ESI) compound 4, m/z: calc. for $C_{31}H_{52}OH^+$: 441,4091 $[M+H]^+$; found: 441,4095.

HRMS (ESI) compound 5, m/z: calc. for $C_{31}H_{52}OH^+$: 441,4091 $[M+H]^+$; found: 441,4093.

Cowan-Mosher-type reduction of lupenone with *n*-BuMgCl

Under argon atmosphere, *n*-BuMgCl (0.35 mL of a solution 2.0 M in THF, 0.7 mmol, 2.0 equiv) was added dropwise to a solution of lupenone (150 mg, 0.35 mmol, 1.0 equiv) in dry THF (5 mL) at −78 °C and the resulting mixture was allowed to slowly warm to room temperature and stirred overnight, before being quenched with 1M HCl (3 mL). The resulting mixture was extracted 3 times with Et_2O, the organic phases were combined, washed with brine, dried over anhydrous Na_2SO_4 and concentrated in vacuo. Compound **1** was obtained in 79% yield (118.0 mg) as white solid after chromatography on silica gel (90:10 v/v, *n*-hexane/ethyl acetate).

3-butyl lupeol (6)

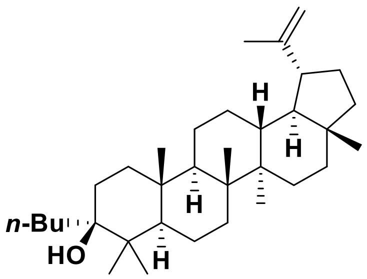

Under argon atmosphere, *n*-BuLi (0.28 mL of a solution 2.5 M in hexane, 0.7 mmol, 2.0 equiv) was added dropwise to a solution of lupenone (150 mg, 0.35 mmol, 1.0 equiv) in dry THF (5 mL) at −78 °C and the resulting mixture was allowed to slowly warm to room temperature and stirred overnight, before being quenched with 1M HCl (3 mL). The resulting mixture was extracted 3 times with Et_2O, the organic phases were combined, washed with brine, dried over anhydrous Na_2SO_4 and concentrated in vacuo. Compound **6** was obtained in 85% yield (143.6 mg) as white solid (m.p.: 157-158 °C) after chromatography on silica gel (95:5 v/v, *n*-hexane/ethyl acetate).

HRMS (ESI), m/z: calc. for $C_{34}H_{58}OH^+$: 483,4560 $[M+H]^+$; found: 483,4563.

3-benzyl lupeol (7) and 3-epi-3-benzyl lupeol (8)

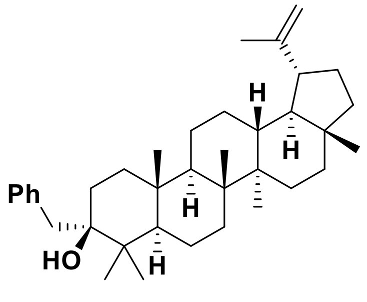

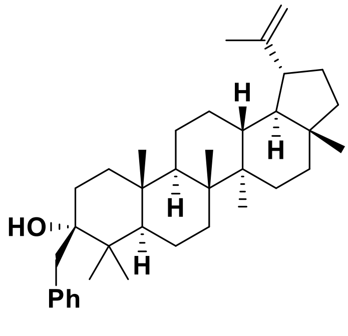

Under argon atmosphere, benzylmagnesium chloride (0.7 mL of a solution 1.0 M in diethyl ether, 0.7 mmol, 2.0 equiv) was added dropwise to a solution of lupenone (150 mg, 0.35 mmol, 1.0 equiv) in dry THF (5 mL) at −78 °C and the resulting mixture was allowed to slowly warm to room temperature and stirred overnight, before being quenched with 1M HCl (3 mL). The resulting mixture was extracted 3 times with Et_2O, the organic phases were combined, washed with brine, dried over anhydrous Na_2SO_4 and concentrated in vacuo. Compound **7** was obtained in 59% yield (107.3 mg) as white solid (m.p.: 181-182 °C) and compound

8 was obtained in 27% yield (48.2 mg) as white solid (m.p.: 169-170 °C) after chromatography on silica gel (96:4 v/v, *n*-hexane/ethyl acetate). Overall yield: 86%, *d.e.*: 38%.

HRMS (ESI) compound 7, m/z: calc. for $C_{37}H_{56}OH^{+}$: 517,4404 $[M+H]^{+}$; found: 517,4401.

HRMS (ESI) compound 8, m/z: calc. for $C_{37}H_{56}OH^{+}$: 517,4404 $[M+H]^{+}$; found: 517,4402.

3-(4-fluorobenzyl)lupeol (9) and 3-epi-3-(4-fluorobenzyl)lupeol (10)

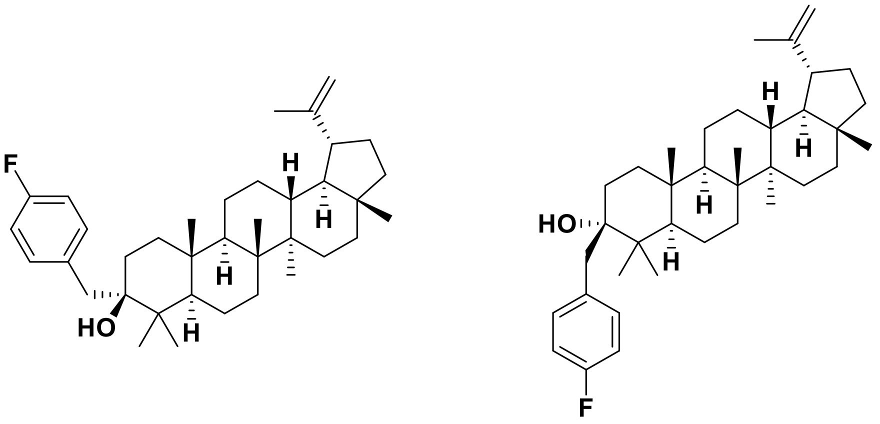

Preparation of 4-fluorobenzylmagnesium bromide: Under argon atmosphere, to a suspension of powdered magnesium metal (35.7 mg, 1.46 mmol, 4.2 equiv) in 4 mL of anhydrous diethyl ether, a drop of 1.2 dibromoethane and a grain of I_2 were added and the red mixture was vigorously stirred until a straw-yellow colour appeared. 4-fluorobenzyl bromide (0.09 mL, 139.9 mg, 0.74 mmol, 2.1 equiv) in dry diethyl ether (2.0 mL) was slowly added over 15 minutes and the final mixture was refluxed for 3 hours. Upon completion, the solution was cooled to 0 °C by ice-water bath and collected by syringe under constant flow of argon.

Under argon atmosphere, the solution of freshly-prepared 4-fluorobenzylmagnesium bromide was added dropwise to a solution of lupenone (150 mg, 0.35 mmol, 1.0 equiv) in dry THF (5 mL) at −78 °C and the resulting mixture was allowed to slowly warm to room temperature and stirred overnight, before being quenched with 1M HCl (3 mL). The resulting mixture was extracted 3 times with Et_2O, the organic phases were combined, washed with brine, dried over anhydrous Na_2SO_4 and concentrated in vacuo.

Compound **9** was obtained in 56% yield (105.3 mg) as white solid (m.p.: 188-189 °C) and compound **10** was obtained in 28 % yield (51.9 mg) as white solid (m.p.: 173-174 °C) after chromatography on silica gel (96:4 v/v, *n*-hexane/ethyl acetate). Overall yield: 84%, *d.e.*: 34%.

HRMS (ESI) compound 9, m/z: calc. for $C_{33}H_{54}FOH^{+}$: 467,4247 $[M+H]^{+}$; found: 467,4244.

HRMS (ESI) compound 10, m/z: calc. for $C_{33}H_{54}FOH^{+}$: 467,4247 $[M+H]^{+}$; found: 467,4242.

3-allyl lupeol (11) and 3-epi-3-allyl lupeol (12)

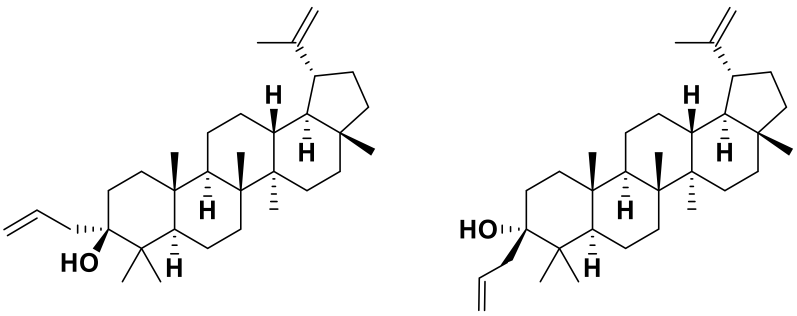

From allylmagnesium bromide: Under argon atmosphere, allylmagnesium bromide (0.7 mL of a solution 1.0 M in diethyl ether, 0.7 mmol, 2.0 equiv) was added dropwise to a solution of lupenone (150 mg, 0.35 mmol, 1.0 equiv) in dry THF (5 mL) at −78 °C and the resulting mixture was allowed to slowly warm to room temperature and stirred overnight, before being quenched with 1M HCl (3 mL). The resulting mixture was extracted 3 times with Et_2O, the organic phases were combined, washed with brine, dried over anhydrous Na_2SO_4 and concentrated in vacuo. Compound **11** was obtained in 61% yield (98.9 mg) as white solid (m.p.: 137-138 °C) and compound **12** was obtained in 28% yield (46.5 mg) as white solid (m.p.: 149-150°C) after chromatography on silica gel (95:5 v/v, *n*-hexane/ethyl acetate). Overall yield: 89%, *d.e.*: 36%.

From allyllithium: Following a previously reported procedure,[3] under argon atmosphere, *n*-BuLi (0.28 mL of a solution 2.5 M in hexane, 0.7 mmol, 2.0 equiv) was added dropwise to a solution of allyltributylstannane (0.23 mL, 245.0 mg, 0.74 mmol, 2.1 equiv) in dry THF (3 mL) at −78 °C and the resulting mixture was stirred for a further 1 h. The solution of freshly-prepared allyllithium was added dropwise to a solution of lupenone (150 mg, 0.35 mmol, 1.0 equiv) in dry THF (5 mL) at −78 °C and the resulting mixture was allowed to slowly warm to room temperature and stirred overnight, before being quenched with 1M HCl (3 mL). The resulting mixture was extracted 3 times with Et_2O, the organic phases were combined, washed with brine, dried over anhydrous Na_2SO_4 and concentrated in vacuo. Compound **11** was obtained in 72% yield (117.5 mg) as white solid (m.p.: 149-150°C) and compound **12** was obtained in 19% yield (31.2 mg) as white solid (m.p.: 137-138 °C) after chromatography on silica gel (95:5 v/v, *n*-hexane/ethyl acetate). Overall yield: 91%, *d.e.*: 58%.

From allylindium bromide: Allylbromide (0.47 mL, 652.1 mg, 5.39 mmol, 15.4 equiv) was added dropwise to a solution of Lupenone (150 mg, 0.35 mmol, 1.0 equiv) and powdered metal indium (618.9 mg, 5.39 mmol, 15.4 equiv) in dry THF (7.5 mL) at room temperature and the solution was stirred for 24 hours at 30 °C. The final mixture was then cooled to 0 °C by ice bath and quenched with 1M HCl (3 mL). The residual metal indium was filtered off, the resulting mixture was extracted 3 times with Et_2O, the organic phases were combined, washed with brine, dried over anhydrous Na_2SO_4 and concentrated in vacuo. Compound **11** was obtained in 25% yield (40.8 mg) as white solid (m.p.: 149-150°C) and compound **12** was obtained in 71% yield (116.1 mg) as white solid (m.p.: 137-138 °C) after chromatography on silica gel (95:5 v/v, *n*-hexane/ethyl acetate). Overall yield: 96%, *d.e.*: 48%.

HRMS (ESI) compound 11, m/z: calc. for $C_{37}H_{55}OH^+$: 535,4310 $[M+H]^+$; found: 535,4308.

HRMS (ESI) compound 12, m/z: calc. for $C_{37}H_{55}OH^+$: 535,4310 $[M+H]^+$; found: 535,4313.

3-(R)-3,1'-epoxy-lup-20-ene (13), 3-(S)-3,1'-epoxy-lup-20-ene (14) and 3-hydroxymethyl-lup-1,20-diene (15)

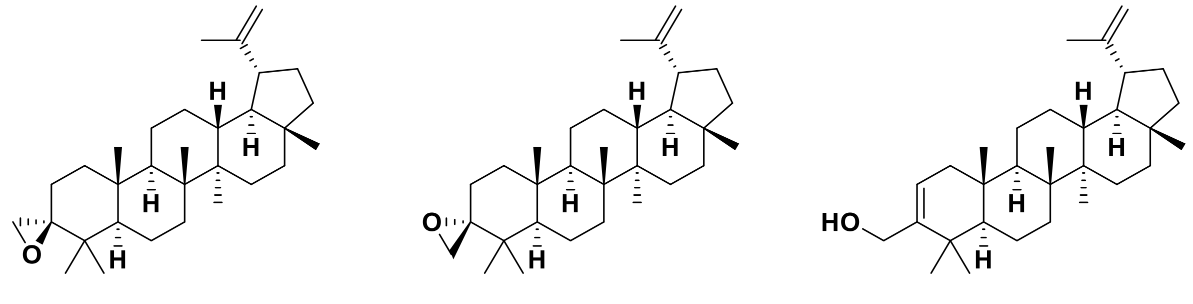

Under argon atmosphere, MeLi-LiBr (0.44 mL of a solution 2.2 M in diethyl ether, 0.98 mmol, 2.8 equiv) was added dropwise to a solution of lupenone (150 mg, 0.35 mmol, 1.0 equiv) and chloroiodomethane (0.08 mL, 185.2 mg, 1.05 mmol, 3.0 equiv) in dry THF (5 mL) at −78 °C and the resulting mixture was allowed to slowly warm to room temperature and stirred overnight, before being quenched with 1M HCl (3 mL). The

resulting mixture was extracted 3 times with Et_2O, the organic phases were combined, washed with brine, dried over anhydrous Na_2SO_4 and concentrated in vacuo. The mixture of compounds **13** and **14** was obtained in 65% yield (99.8 mg) as white solid (m.p.: range 165-174 °C) (ratio **13**/**14** calculated from ^{1}H-NMR: 1:0.17) and compound **15** was obtained in 18% yield (27.6 mg) as white solid (m.p.: 248 °C) after chromatography on silica gel (gradient from 95:5 to 90:10 v/v, *n*-hexane/ethyl acetate). Overall yield: 83%.
HRMS (ESI) compounds 13 and 14, m/z: calc. for $C_{31}H_{50}OH^+$: 439,3934 $[M+H]^+$; found: 439,3939.
HRMS (ESI) compound 15, m/z: calc. for $C_{31}H_{50}OH^+$: 439,3934 $[M+H]^+$; found: 439,3932.

30-formyl lupeol (3)

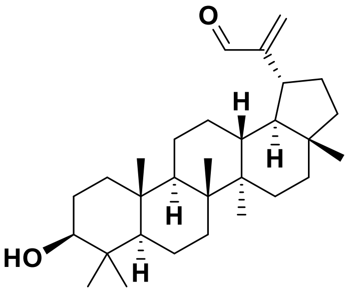

A solution of lupeol (500 mg, 1.17 mmol, 1.0 equiv) and SeO_2 (194.2 mg, 1.75 mmol, 1.5 equiv) in dry ethanol (20 mL) was refluxed for 48 hours. The resulting mixture was filtered through celite, extracted 3 times with Et_2O, the organic phases were combined, washed with brine, dried over anhydrous Na_2SO_4 and concentrated in vacuo. Compound **3** was obtained in 54% yield (278.43 mg) as white solid after chromatography on silica gel (85:15 v/v, *n*-hexane/ethyl acetate). Melting point and NMR data correspond to those reported in the literature.[1a,4]

1',30-epoxy-lupeol (16)

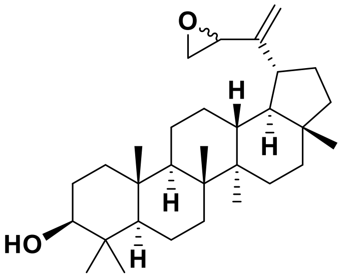

Under argon atmosphere, MeLi-LiBr (0.34 mL of a solution 2.2 M in diethyl ether, 0.76 mmol, 2.8 equiv) was added dropwise to a solution of 30-formyl-lupeol (**15**) (120 mg, 0.27 mmol, 1.0 equiv) and chloroiodomethane (0.06 mL, 142.9 mg, 0.81 mmol, 3.0 equiv) in dry THF (5 mL) at −78 °C and the resulting mixture was allowed to slowly warm to room temperature and stirred overnight, before being quenched with 1M HCl (3 mL). The resulting mixture was extracted 3 times with Et_2O, the organic phases were combined, washed with brine, dried over anhydrous Na_2SO_4 and concentrated in vacuo. Compounds **16** was obtained in 83% yield (101.9 mg) as white solid (m.p.: 198-212 °C) (ratio calculated from ^{1}H-NMR 1:0.6) after chromatography on silica gel (90:10 v/v, *n*-hexane/ethyl acetate).
HRMS (ESI), m/z: calc. for $C_{31}H_{50}O_2H^+$: 455,3884 $[M+H]^+$; found: 455,3888

1'-chloro-30-hydroxy-lupeol (17)

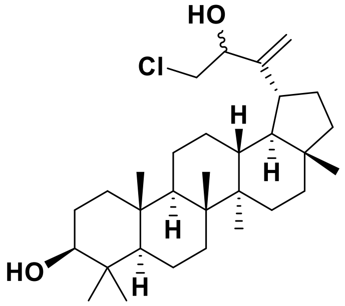

Under argon atmosphere, MeLi-LiBr (0.34 mL of a solution 2.2 M in diethyl ether, 0.76 mmol, 2.8 equiv) was added dropwise to a solution of 30-formyl-lupeol (**15**) (120 mg, 0.27 mmol, 1.0 equiv) and chloroiodomethane (0.06 mL, 142.9 mg, 0.81 mmol, 3.0 equiv) in dry THF (5 mL) at −78 °C and the resulting mixture was stirred at low temperature for 1 hour, before being quenched with 1M HCl (3 mL). The resulting mixture was extracted 3 times with Et_2O, the organic phases were combined, washed with brine, dried over anhydrous Na_2SO_4 and concentrated in vacuo. Compounds **17** was obtained in 86% yield (114.1 mg) as white solid (m.p.: 241-253 °C) (ratio calculated from ^{1}H-NMR spectrum of the crude reaction mixture: 1:0.6) after chromatography on silica gel (85:15 v/v, *n*-hexane/ethyl acetate).
HRMS (ESI), m/z: calc. for $C_{31}H_{51}ClO_2H^+$: 491,3650 $[M+H]^+$; found: 491,3647.

Full ^{1}H- and ^{13}C-NMR assignment for all the products

	3-epi-3-methyl-lupeol (5)			3-methyl-lupeol (4)		
Position	^{13}C (ppm)	^{1}H (ppm)	J (MHz)	^{13}C (ppm)	^{1}H (ppm)	J (MHz)
1	32.5 t	1.34 m,<br>1.82 m		38.0 t	1.00 m<br>1.56 dt	 13.4, 3.6
2	35.7	1.42 m,<br>1.46 m		34.6 t	1.31 m<br>1.36 m	
3	74.6	-		75.4 s	-	
4	37.5	-		37.6 s	-	
5	50.8	1.22 m		53.5 s	0.91 br-d	8.9
6	18.9	1.35 m,<br>1.43 m		19.0 t	1.37 m<br>1.45 m	
7	34.4	1.34 m,<br>1.38 m		34.3 t	1.78 m<br>1.84 td	 13.7, 3.9
8	40.9	-		41.0 s	-	
9	50.5	1.38 m		51.0 d	1.30 m	
10	40.5	-		41.0 s	-	
11	20.9	1.20 m,<br>1.44 m		21.0 t	1.19 m,<br>1.40 m	
12	25.3	1.64 m,<br>1.67 m		25.3 t	1.65 m,<br>1.68 m	
13	38.2	1.65 m		38.2 d	1.65 m	
14	43.0	-		42.9 s	-	
15	27.6	0.99 dt<br>1.06 dd	13.6, 2.5<br>13.1, 4.5	27.6 t	0.99 dt<br>1.07 dd	13.6, 2.7<br>12.9, 4.7
16	35.1	1.18 m,<br>1.24 m		35.7 t	1.47 m<br>1.49 m	
17	43.2	-		43.1 s	-	
18	48.4	1.36 m		48.1 d	1.35 m	
19	48.2	2.37 td	11.1, 5.9	48.4 d	2.37 td	11.1, 5.9
20	151.2	-		151.1 s	-	
21	30.0	1.28 m<br>1.91 m		30.0 t	1.25 m<br>1.91 m	
22	40.2	1.17 m<br>1.38 m		40.1 t	1.17 m<br>1.37 m	
23	23.9	0.90 s		19.1 q	0.86 s	
24	21.1	0.77 s		24.6 q	0.84 s	
25	15.8	0.80 s		16.2 q	1.02 s	

26	16.1	1.02 s		16.5 q	0.84 s	
27	14.8	0.95 s		14.8 q	0.95 s	
28	18.2	0.78 s		18.1 q	0.78 s	
29	109.5	4.67 d,<br>4.55 m	2.4	109.5 t	4.68 d,<br>4.56 m	2.2
30	19.4	1.67 s		19.4 q	1.68 s	
1’	25.6	1.10 s		23.4 q	1.19 s	

	**3-butyl-lupeol (6)**		
Position	^{13}C (ppm)	^{1}H (ppm)	J (MHz)
1	37.2	1.46 m,<br>1.51 m	
2	29.8	1.23 m,<br>1.31 m	
3	76.6	-	
4	37.5	-	
5	53.1	0.95 m	
6	19.1	1.37 m,<br>1.41 m	
7	34.6	1.37 m,<br>1.38 m	
8	41.7	-	
9	51.0	1.27 m	
10	41.0	-	
11	21.0	1.36 m,<br>1.41 m	
12	25.2	1.62 m,<br>1.67 m	
13	38.1	1.64 m	
14	42.9	-	
15	27.6	1.24 m,<br>1.51 m	
16	35.7	1.22 m,<br>1.48 m	
17	43.1	-	
18	48.4	1.35 m	
19	48.1	2.36 td	11.1, 5.8

20	151.0	-	
21	29.9	1.31 m,<br>1.91 m	
22	40.1	1.17 m,<br>1.38 m	
23	23.6	0.80 s	
24	19.3	0.85 s	
25	15.9	1.02 s	
26	16.6	0.85 s	
27	14.4	0.95 s	
28	18.1	0.98 s	
29	109.4	4.55 m,<br>4.67 d	2.4
30	19.3	1.66 s	
1’	32.7	1.29 –<br>1.31 m	
2’	27.8	1.50 –<br>1.53 m	
3’	23.9	1.32 m	
4’	14.8	0.92 t	7.0

	**3-epi,3-benzyl lupeol (8)**			**3-benzyl lupeol (7)**		
Position	^{13}C (ppm)	^{1}H (ppm)	J (MHz)	^{13}C (ppm)	^{1}H (ppm)	J (MHz)
1	34.6 t	1.46 m<br>1.48 m		37.9 t	1.48 m<br>1.12 m	
2	29.1 t	1.15 m<br>1.74 m		28.3 t	0.99 m<br>1.17 m	
3	75.1 s	-		76.5 s		
4	37.2 s	-		37.5 s		
5	51.2 d	1.35 m		53.3 d	1.10 d br	8.8
6	19.0 t	1.5 m<br>1.50 m		19.1 t	1.45 m<br>1.51 m	
7	34.4 t	1.02 m<br>1.34 m		34.6 t	1.40 m<br>1.46 m	
8	40.9 s	-		41.6 s		
9	50.4 d	1.34 m		51.3 d	1.40 m	
10	40.8 s	-		41.0 s		

11	20.8 t	1.17 m<br>1.38 m		21.1 t	1.13 m<br>1.44 m	
12	25.2 t	1.03 m<br>1.63 m		25.3	1.67 m<br>1.73 m	
13	38.2 d	1.65 m		38.2	1.68 m	
14	43.0 s	-		43.0		
15	27.5 t	1.02 m<br>1.67 m		27.6	1.26 m<br>1.74 m	
16	35.7 t	1.05 m<br>1.45 m		35.7	1.50 m<br>1.70 m	
17	43.1 s	-		43.1		
18	48.4 d	1.35 m		48.5	1.39 m	
19	48.1 d	2.37 td	11.1, 6.0	48.5	2.39 td	15.5, 5.9
20	151.1 s	-		151.0		
21	30.0 t	1.31m<br>1.91 m		30.0	1.28 m<br>1.92 m	
22	40.1 t	1.17 m<br>1.38 m		40.1	1.20 m<br>1.40 m	
23	23.8 q	1.05 s		24.6	0.99 s	
24	20.8 q	0.91 s		19.9	0.92 s	
25	15.8 q	0.84 s		16.1	1.06 s	
26	16.1 q	1.04 s		16.8	0.88 s	
27	14.7 q	0.94 s		14.8	1.02 s	
28	18.1 q	0.79 s		18.1	0.80 s	
29	109.4 t	4.54 s br<br>4.67 s br		109.4	4.57 m<br>4.70 d br	2.40
30	19.4 q	1.66 s		19.5	1.70 s	
1’	42.4 t	2.53 d<br>2.98 d	13.4<br>13.0	38.5 t	2.81 d br<br>2.98 d	13.6<br>13.6
2’	137.9 s			138.6 s		
3’, 7’	131.3 d	7.20 m		130.9 d	7.27 m	
4’, 6’	128.1 d	7.30 m		128.1 d	7.28 m	
5’	126.0 d	7.23 m		126.3 d	7.22 m	

	3-epi, 3-(4-fluorobenzyl)lupeol (10)			3-(4-fluorobenzyl)lupeol (9)		
Position	^{13}C (ppm)	^{1}H (ppm)	J (MHz)	^{13}C (ppm)	^{1}H (ppm)	J (MHz)
1	34.5	1.35 m<br>1.44 m		37.9	1.07 m<br>1.51 m	
2	29.0	1.12 m<br>1.68 m		28.2	1.09 m<br>1.46 m	
3	75.0	-		76.5	-	
4	37.1	-		37.5	-	
5	51.1	1.30 m		53.3	1.08 m	
6	18.9	1.37 m<br>1.47 m		19.1	1.45 m<br>1.51 m	
7	34.3	0.90 m<br>1.34 m		34.6	1.35 m<br>1.43 m	
8	40.9	-		41.6	-	
9	50.4	1.33 m		51.2	1.39 m	
10	40.8	-		41.0	-	
11	20.8	1.17 m<br>1.36 m		21.1	1.24 m<br>1.42 m	
12	25.2	0.96 m<br>1.63 m		25.3	0.90 m<br>1.70 m	
13	38.1	1.63 m		38.1	1.68 m	
14	42.9	-		43.0	-	
15	27.5	0.96 m<br>1.64 m		27.6	1.00 m<br>1.70 m	
16	35.6	1.20 m<br>1.48 m		35.7	1.38 m<br>1.51 m	
17	43.1	-		43.1	-	
18	48.3	1.34 m		48.4	1.40 m	
19	48.1	2.36 td	11.0, 5.8	48.1	2.39 td	11.0, 5.7
20	151.1	-		151.0	-	
21	29.9	1.30 m<br>1.90 m		29.9	1.33 m<br>1.92 m	
22	40.1	1.17 m<br>1.37 m		40.1	1.20 m<br>1.41 m	
23	23.8	1.01 s		24.5	0.97 s	
24	20.7	0.89 s		19.9	0.92 s	
25	15.8	0.83 s		16.1	1.05 s	
26	16.1	1.02 s		16.8	0.87 s	
27	14.7	0.93 s		14.8	1.01 s	
28	18.1	0.77 s		18.1	0.80 s	

29	109.4	4.54 s br<br>4.66 s br		109.5	4.58 s br<br>4.70 s br	
30	19.3	1.65 s		19.5	1.70 s	
1’	41.5	2.50 d<br>2.95 d	13.3<br>13.3	37.7	2.78 d<br>2.93 d	13.7<br>13.7
2’ (ipso)	132.6	-		132.2	-	
3’ (o)	133.4	7.14 m		134.3	7.23 m	
4’ (m)	115.0	6.97 m		114.8	6.96 m	
5’ (p)	161.8 d (C-F)	-		161.7	-	

	**3-epi-3-allyl lupeol (10)**			**3-allyl lupeol (11)**		
Position	^{13}C (ppm)	^{1}H (ppm)	J (MHz)	^{13}C (ppm)	^{1}H (ppm)	J (MHz)
1	36.6	1.17 m<br>1.38 m		37.6	1.00 m<br>1.47 m	
2	29.0	1.44 m<br>1.64 m		28.7	1.46 m<br>1.63 m	
3	75.0	-		76.1	-	
4	40.8	-		41.2	-	
5	51.0	1.29 m		53.1	0.97 m	
6	20.8	0.92 m<br>1.20 m		18.9	1.02 m<br>1.68 m	
7	34.3	1.38 m<br>1.46 m		34.5	1.38 m<br>1.61 m	
8	41.0	-		40.8	-	
9	50.3	1.37 m		50.9	1.30 m	
10	37.0	-		37.1	-	
11	20.6	1.20 m<br>1.41 m		20.9	1.21 m<br>1.38 m	
12	25.2	1.08 m<br>1.67 m		25.1	1.09 m<br>1.67 m	
13	38.1	1.66 m		38.0	1.65 m	
14	42.9	-		43.0	-	
15	27.4	0.99 m<br>1.02 m		27.5	1.01 m<br>1.68 m	
16	35.6	1.65 m<br>1.47 m		35.6	1.47 m<br>1.48 m	
17	43.0	-		42.8	-	
18	48.3	1.37 m		48.3	1.36 m	

19	48.0	2.38 ddd	5.9,11.0<br>18.0	48.0	2.38 dt	11.0, 5.8
20	151.0	-		150.9	-	
21	29.9	1.30 m<br>1.91 m		29.8	1.29 m<br>1.90 m	
22	40.0	1.18 m<br>1.37 m		40.0	1.19 m<br>1.38 m	
23	23.6	0.92 s		24.1	0.86 s	
24	18.0	0.81 s		18.9	0.89 s	
25	15.7	0.83 s		16.6	0.87 s	
26	16.0	1.04 s		16.0	1.04 s	
27	14.7	0.97 s		14.7	0.97 s	
28	18.8	0.80s		18.0	0.80 s	
29	109.3	4.57 m<br>4.68 d br	 2.5	109.3	4.56 sex<br>4.69 d br	1.4<br>2.6
30	19.3	1.69 s		19.3	1.69 s	
1’	40.7	2.07 dd br<br>2.40 dd	13.8, 7.6<br>13.9, 7.4	37.3	2.21 dddd<br>2.52 dd	14.1, 6.9, 2.9, 1.5<br>14.1, 7.9
2’	134.8	5.92 ddt	17.0, 10.9, 7.4	135.0	5.90 m	
3’	118.8	5.11 d<br>5.17 dd br.	17.0<br>10.1, 2.3	118.0	5.12 m<br>5.15 m	

	**3-(R)-3,1’-epoxy-lup-20-ene (13)**			**3-hydroxymethyl-lup-1,20-diene (15)**		
Position	^{13}C (ppm)	^{1}H (ppm)	J (MHz)	^{13}C (ppm)	^{1}H (ppm)	J (MHz)
1	38.1	1.65 m<br>1.67 m		41.7	2.02 d br<br>2.07 d br	6.3
2	27.8	0.97 m<br>1.03 m		121.7	5.62 d	5.7
3	64.3	-		144.2	-	
4	37.1	-		36.2	-	
5	56.6	0.91 m		53.1	1.05 m	
6	19.7	1.40 m<br>1.44 m		19.5	1.41 m<br>1.48 m	
7	33.6	1.38 m		33.6	1.38 m	

		1.40 m			1.43 m	
8	40.3	-		40.9	-	
9	50.4	1.34 m		49.5	1.31 m	
10	37.6	-		36.7	-	
11	21.1	0.93 m<br>0.95 m		21.4	0.90 m<br>0.95 m	
12	25.0	1.04 m<br>1.65 m		25.4	1.03 m<br>1.65 m	
13	39.3	1.75 dt	12.9<br>3.8	38.4	1.68 m	
14	43.1	-		43.2	-	
15	27.2	1.68 m<br>2.22 m		27.6	1.04 m<br>1.67 m	
16	35.3	1.31 m<br>1.47 m		35.7	1.34 m<br>1.48 m	
17	42.9	-		42.9	-	
18	48.4	1.35 m		48.4	1.37 m	
19	47.9	2.38 m		48.1	2.39 m	
20	151.1	-		151.1	-	
21	29.6	1.23 m<br>1.92 m		29.8	1.24 m<br>1.92 m	
22	40.1	1.17 m<br>1.38 m		40.1	1.23 m<br>1.33 m	
23	24.4	0.67 s		29.2	1.02 s	
24	19.7	0.93 s		21.1	0.95 s	
25	14.7	0.95 s		14.5	0.83 s	
26	16.0	1.05 s		15.8	1.04 s	
27	16.2	0.93 s		16.5	0.81 s	
28	17.8	0.79 s		18.2	0.80 s	
29	109.3	4.69 s br<br>4.56 s br		109.4	4.57 s br<br>4.69 s br	
30	19.2	1.68		19.5	1.68 s	
1’	52.3	2.37 d br<br>2.88 dd	4.84<br>4.8<br>1.9	63.4	4. 11 d br<br>4.15 d br	13.0

	1’,30-epoxy-lupeol (16) (majority product)			1’,30-epoxy-lupeol (16) (minority product)		
Position	^{13}C (ppm)	^{1}H (ppm)	J (MHz)	^{13}C (ppm)	^{1}H (ppm)	J (MHz)
1	39.0			38.8		
2	27.5			27.5		
3	79.1	3.17 dd	11.5, 4.7	79.1	3.17 dd	11.5, 4.7
4	38.9	-		38.8		
5	55.3	0.67 d br	9.8	55.4	0.67 d br.	9.8
6	18.4			18.4		
7	34.4			34.4		
8	40.9	-		40.9		
9	50.4	1.24 m		50.5	1.20 m	
10	37.3	-		37.3		
11	21.1			21.1		
12	28.1			28.1		
13	38.0			38.1		
14	43.1	-		43.1		
15	26.9			27.2		
16	35.5			35.6		
17	42.8	-		42.9		
18	49.5	1.45 m		50.5	1.54 m	
19	40.2	2.12 m		43.1	2.31 m	
20	151.3	-		151.8		
21	29.8			31.6		
22	40.0	1.27 m<br>1.37 m		40.0	1.20 m<br>1.42 m	
23	28.1	0.96 s		28.1	0.94 s	
24	15.5	0.74 s		15.5	0.74 s	
25	16.2	0.78 s		16.2	0.79 s	
26	16.2	1.01s		16.1	1.02 s	
27	14.6	0.93s		14.6	0.92 s	
28	17.9	0.81s		17.9	0.82 s	
29	110.6	5.03 s Br<br>4.98 s br		108.3	4.88 s br<br>4.84 s br	
30	53.3	3.36 t		52.2	3.28 t	3.3

		3.3				
1’	48.4	2.98 t<br>2.59 dd	4.8<br>5.5, 2.6	50.1	2.92 dd<br>2.56 dd	6.1, 2.4<br>6.2, 2.4

	**1’-chloro-30-hydroxy-lupeol (17)**		
Position	^{13}C (ppm)	^{1}H (ppm)	J (MHz)
1	38.7	1.60 m, 1.66 m	
2	27.5	1.09 m, 1.19 m	
3	79.1	3.17 dd	4.7, 11.6
4	38.9	-	
5	55.3	0.66 m d br	9.3
6	18.4	1.35 m, 1.51 m	
7	34.4	1.36 m, 1.37 m	
8	40.9	-	
9	50.3	1.25 m	
10	37.2	-	
11	21.2	1.22 m, 1.43 m	
12	27.9	0.78 m, 0.89 m	
13	38.0	0.91 m	
14	42.9	-	
15	27.4	1.58 m, 1.68 m	
16	35.4	1.48 m, 1.53 m	
17	43.1	-	
18	52.3	1.46 m	
19	42.9	1.98 m	
20	154.8	-	
21	29.9	1.23 m, 1.24 m	
22	40.0	1.26 m, 1.32 m	
23	28.1	0.95 s	
24	15.5	0.74 s	
25	16.2	0.81 s	
26	16.0	1.01 s	

27	14.5	0.92 s	
28	17.9	0.76 s	
29	74.9	4.22 d br	8.2
30	109.3	5.07 s br<br>5.15 s br	
1’	49.5	3.47 dd<br>3.75 dd	11.3, 8.0<br>11.3, 2.7

Copies of ^{1}H- and ^{13}C-NMR Spectra for all the products

3-methyl lupeol (4)

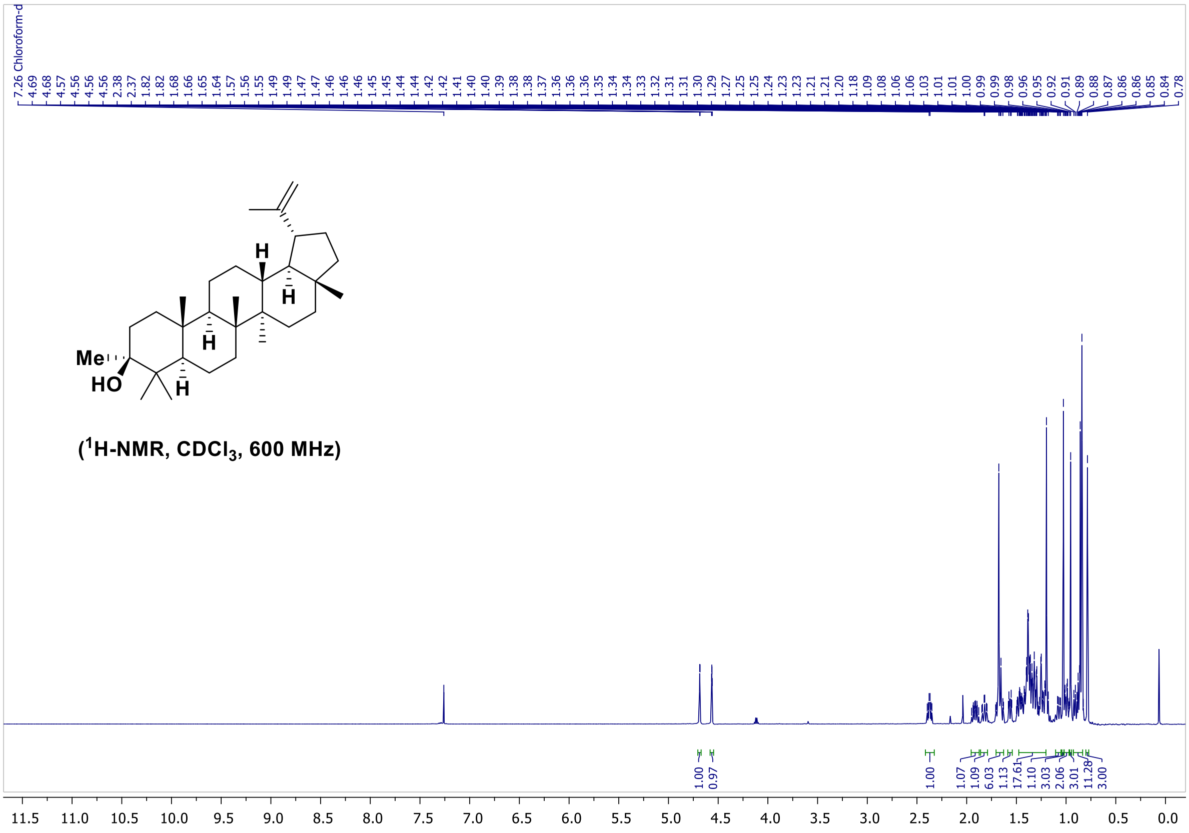

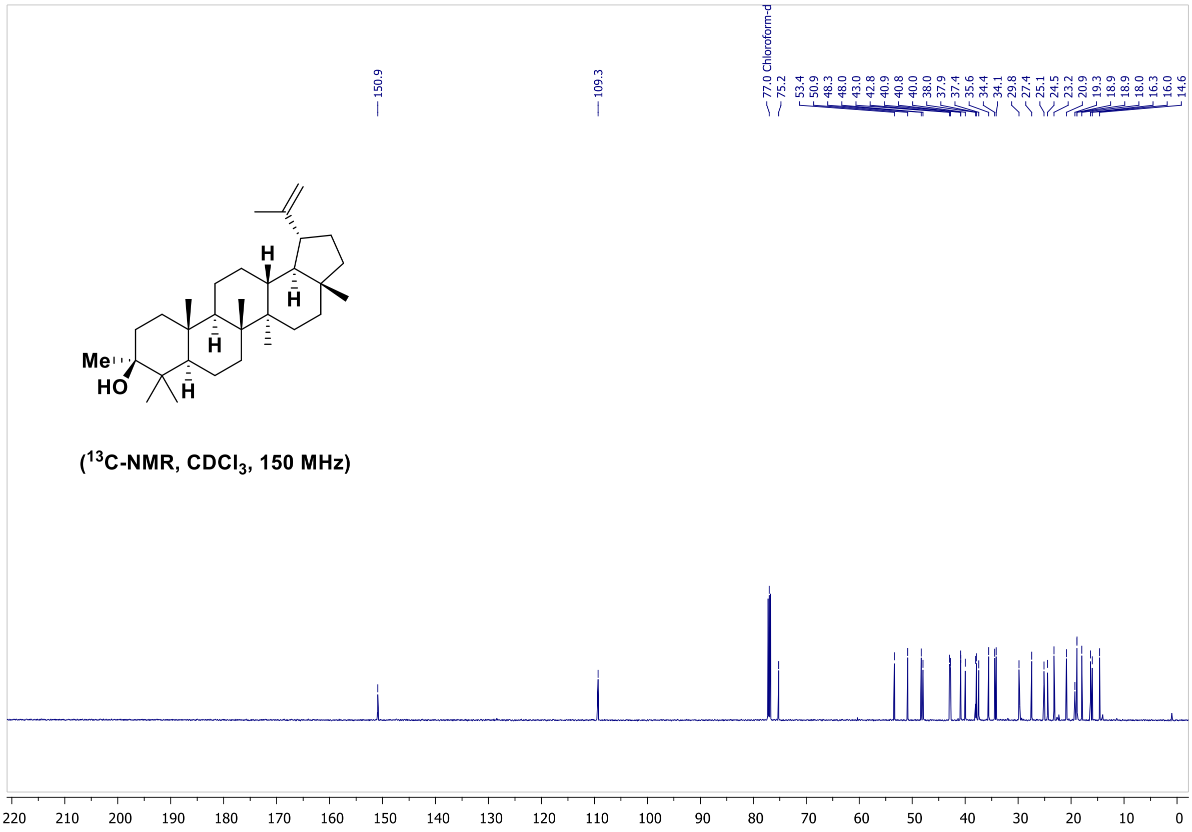

3-epi-3-methyl lupeol (5)

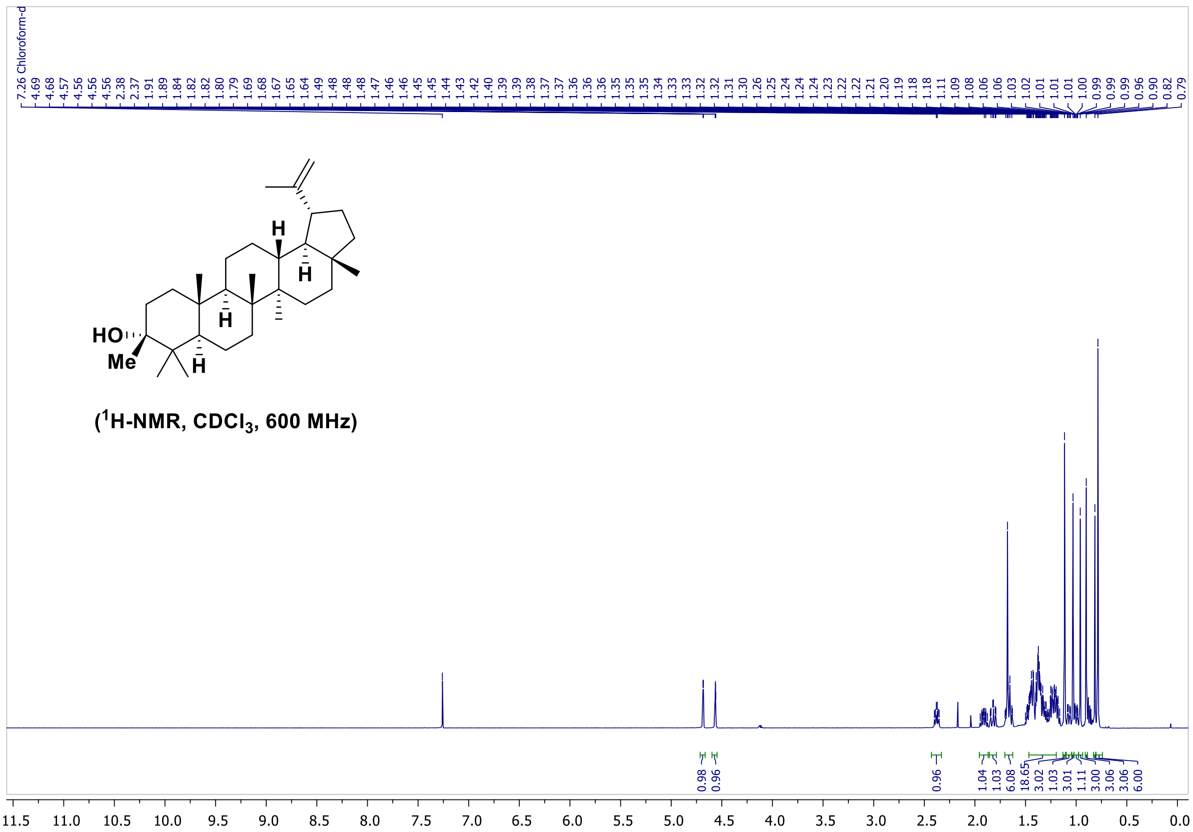

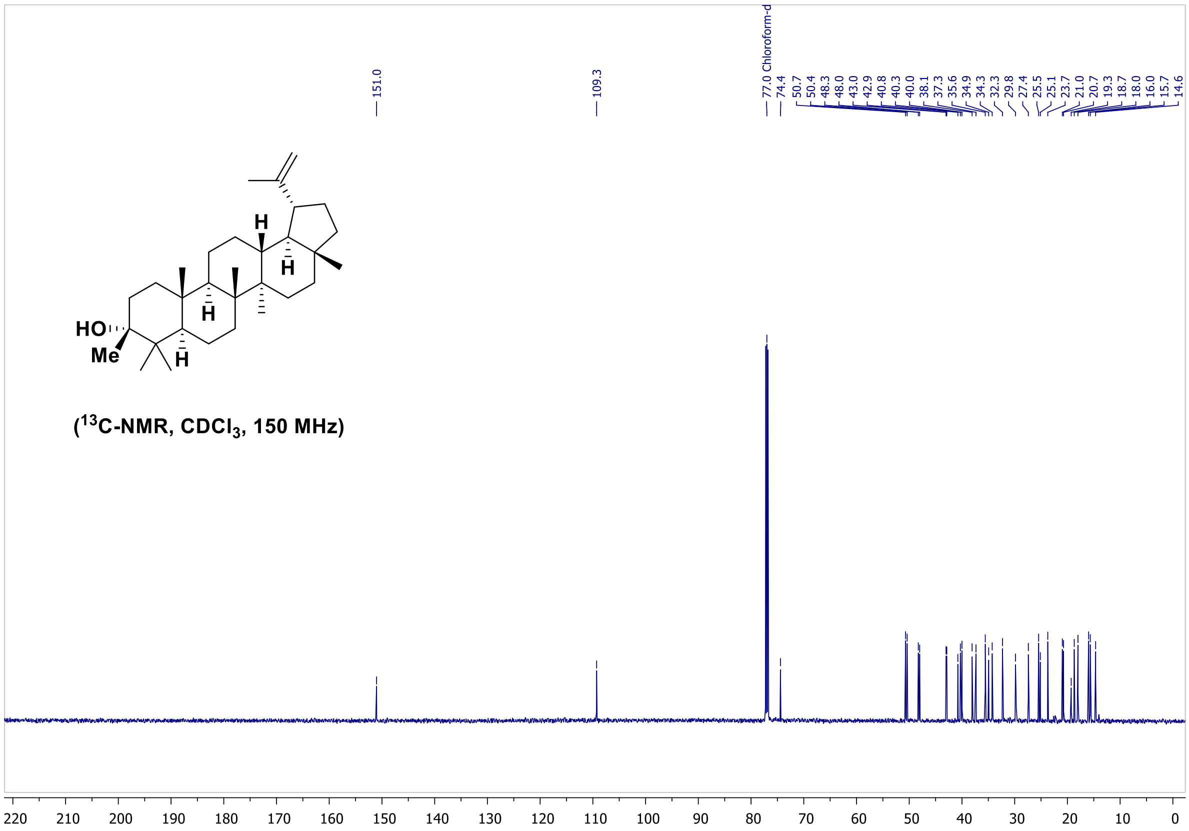

3-butyl lupeol (6)

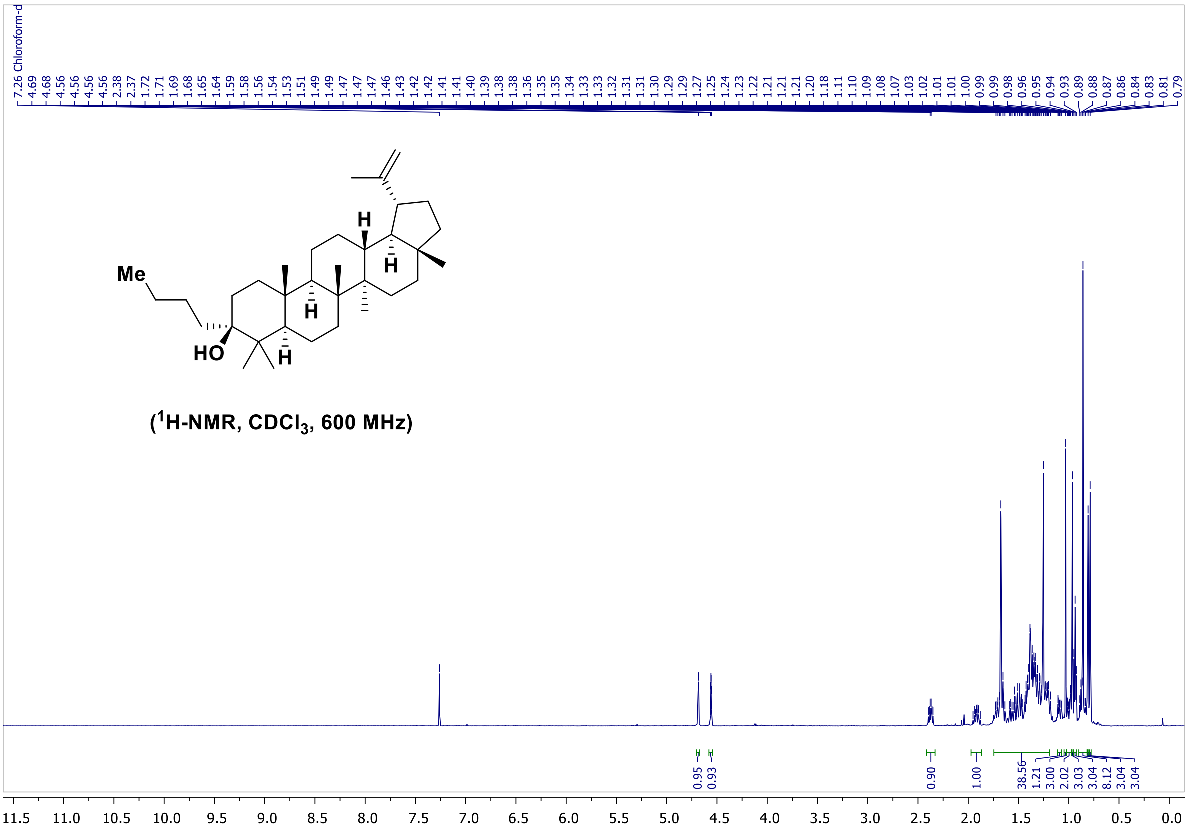

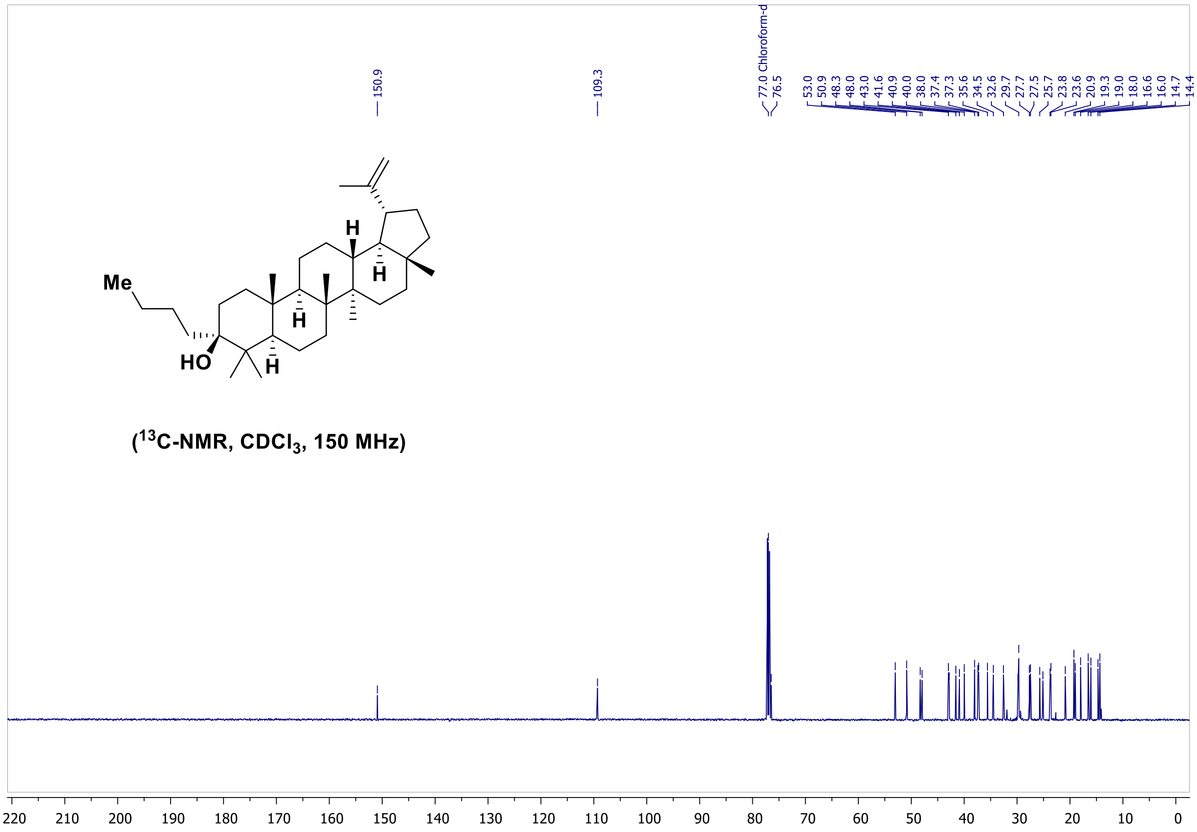

3-benzyl lupeol (7)

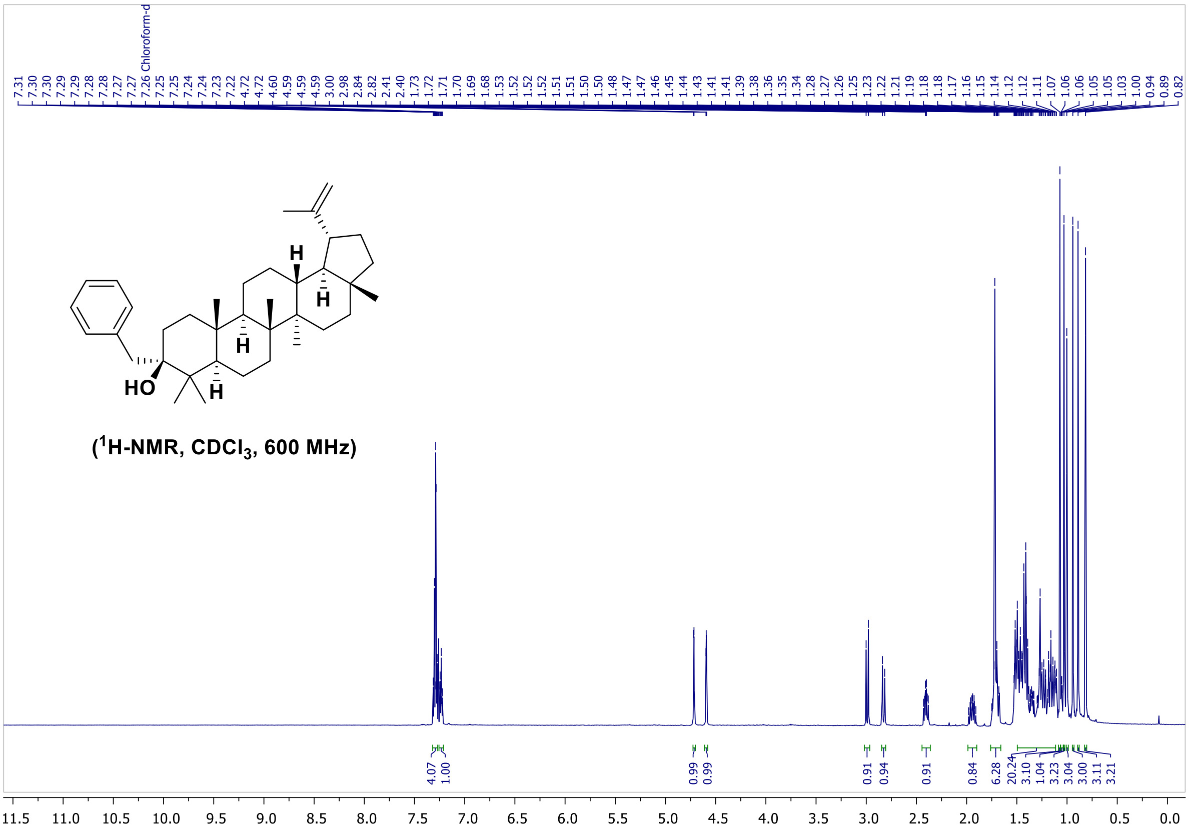

(^{1}H-NMR, $CDCl_3$, 600 MHz)

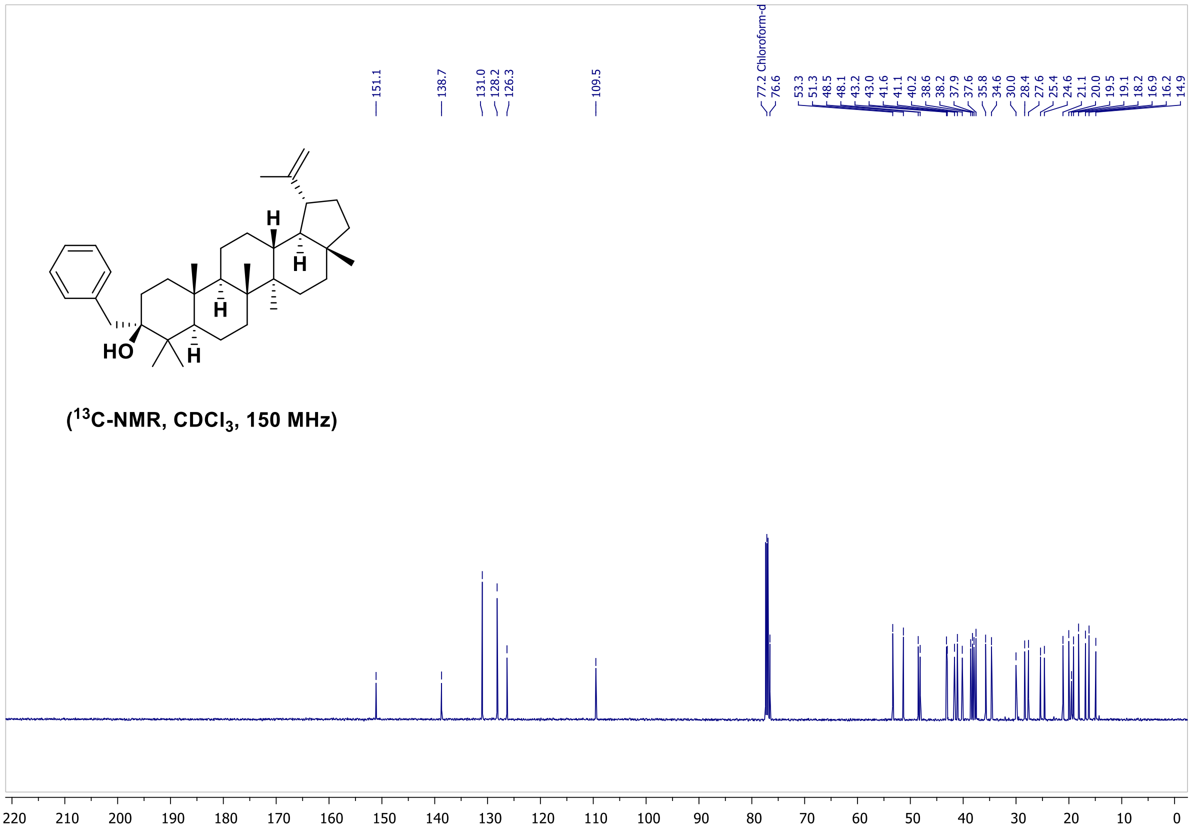

(^{13}C-NMR, $CDCl_3$, 150 MHz)

3-epi-3-benzyl lupeol (8)

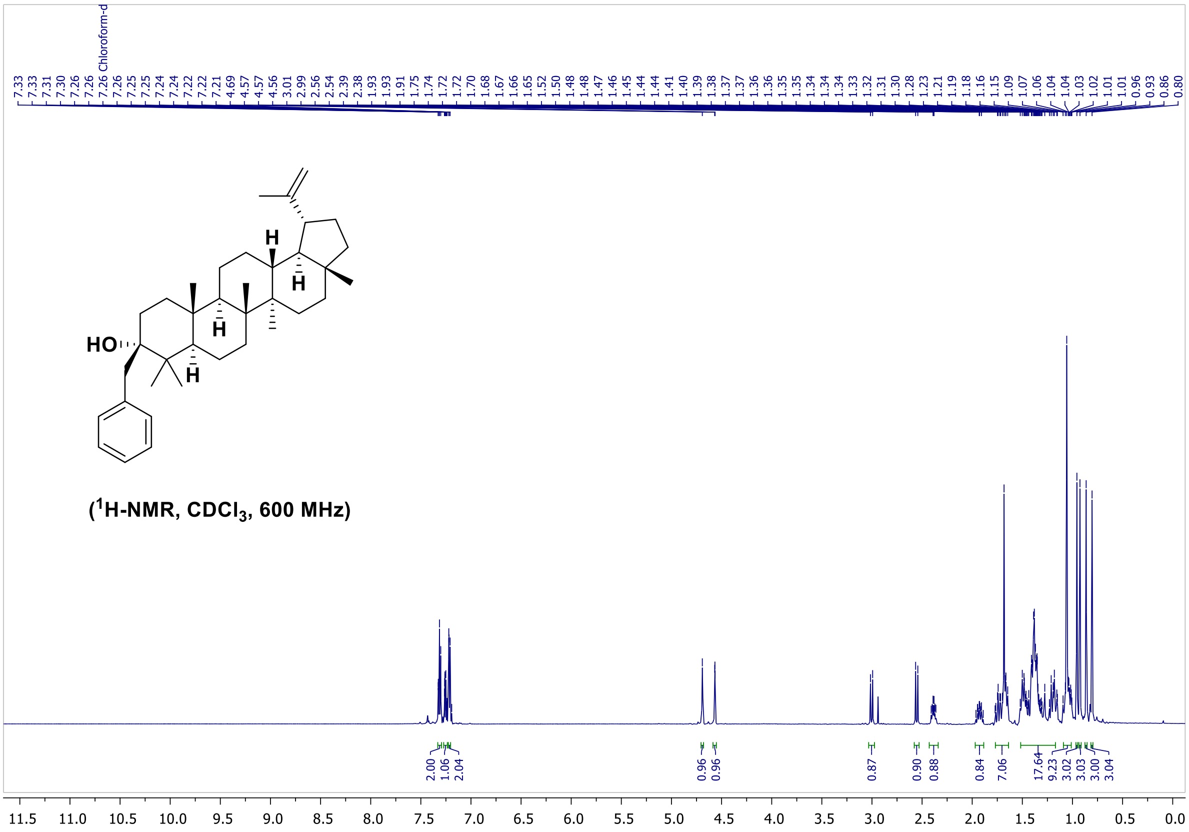

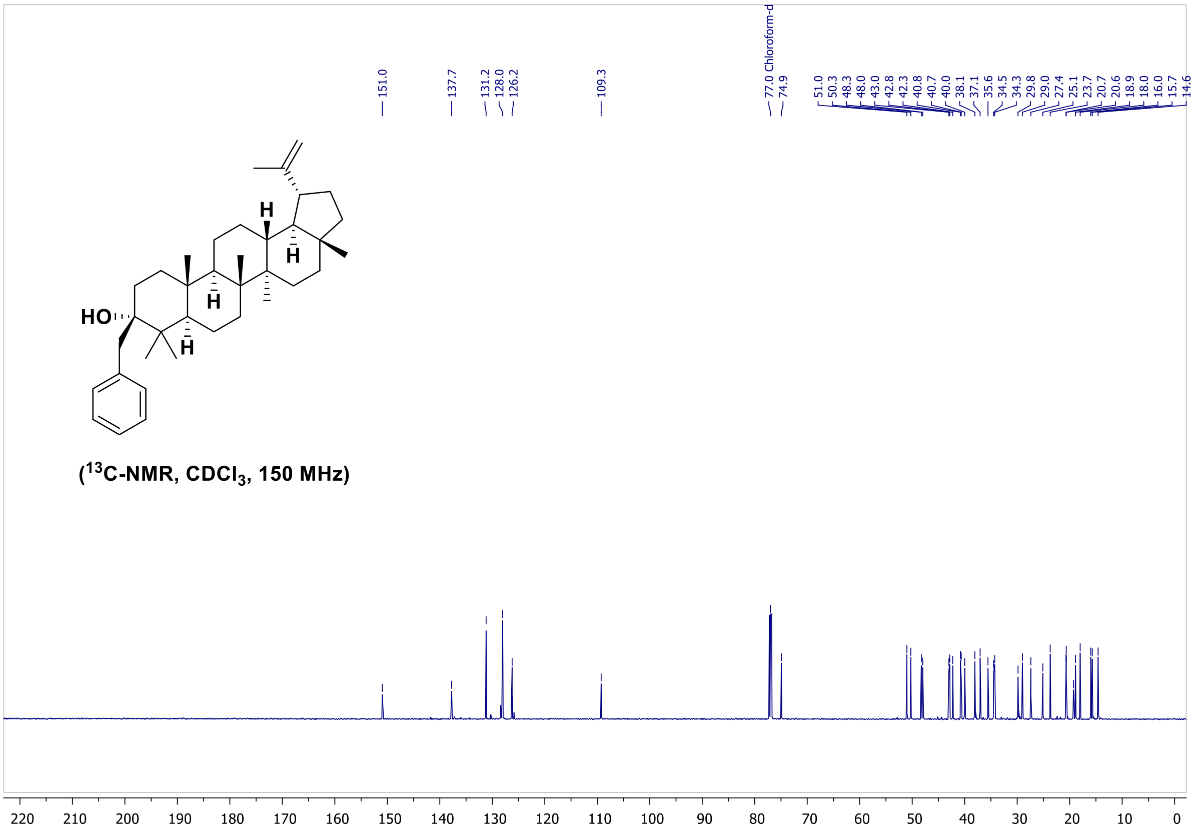

3-(4-fluorobenzyl) lupeol (9)

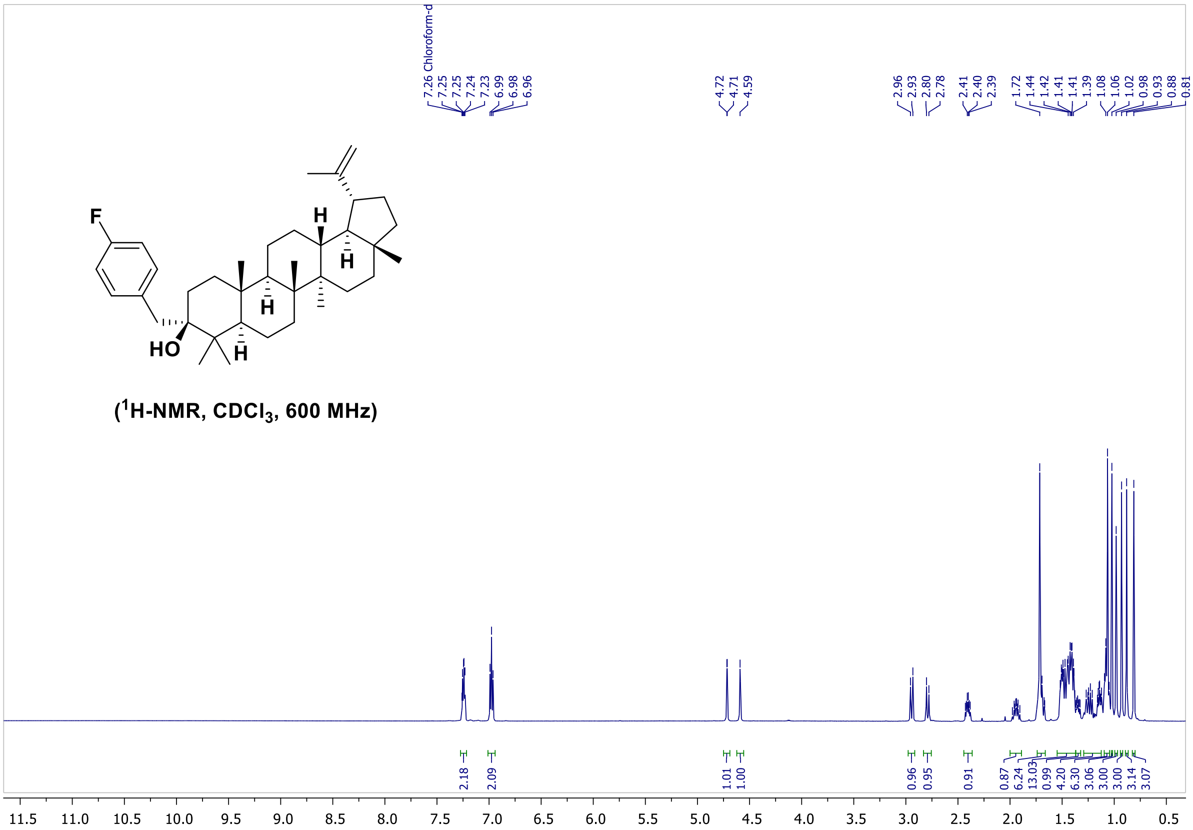

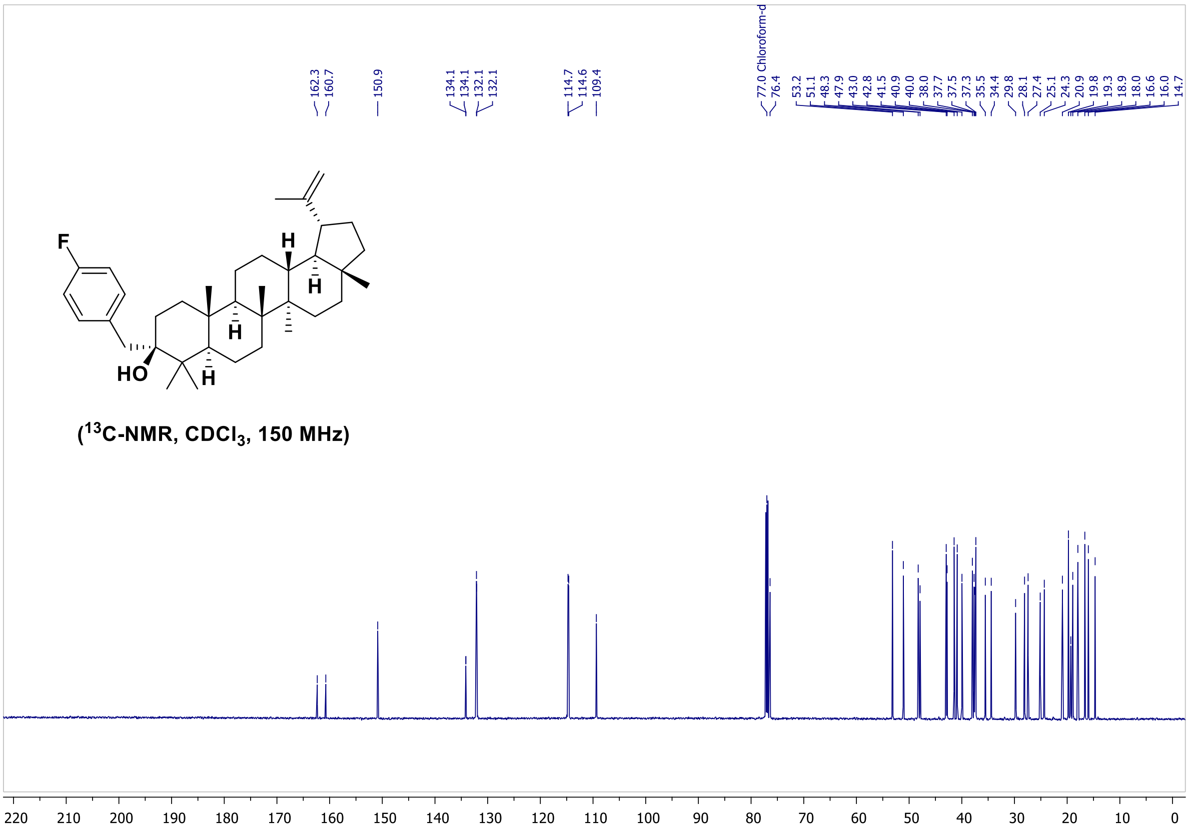

3-epi-3-(4-fluorobenzyl) lupeol (10)

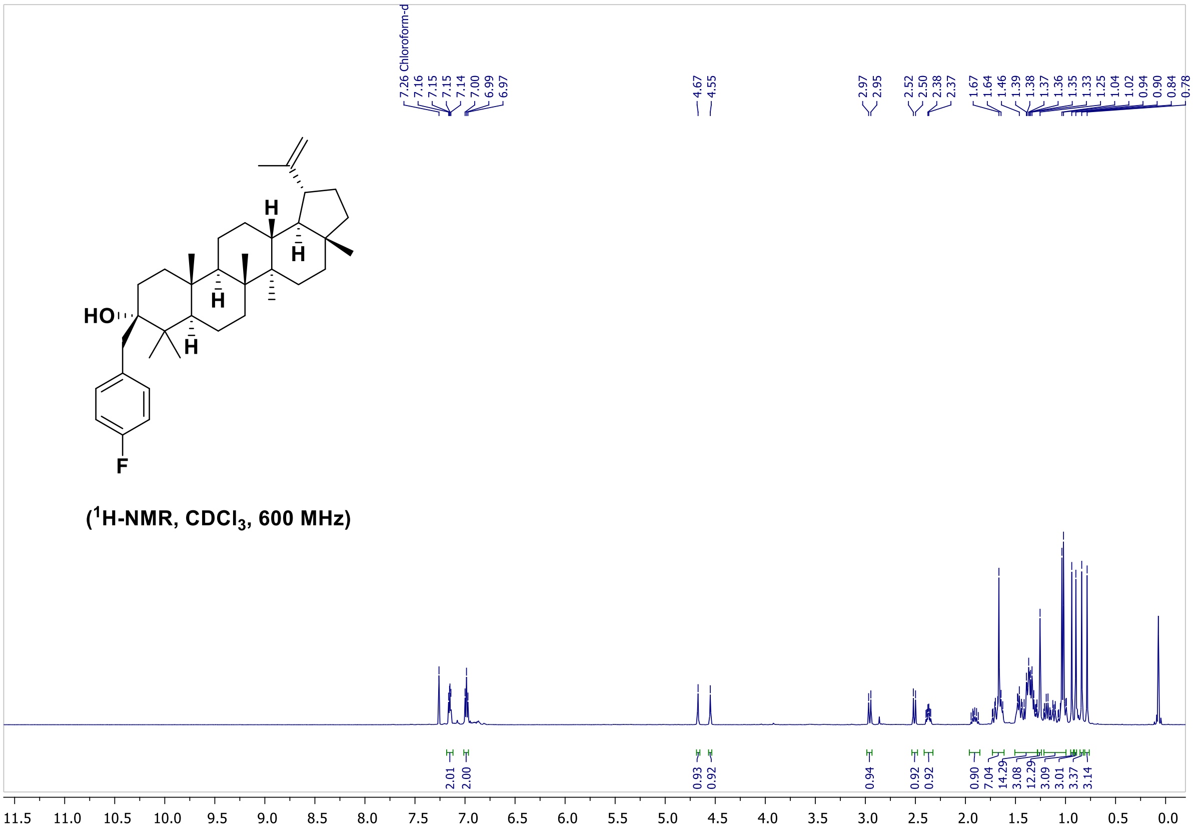

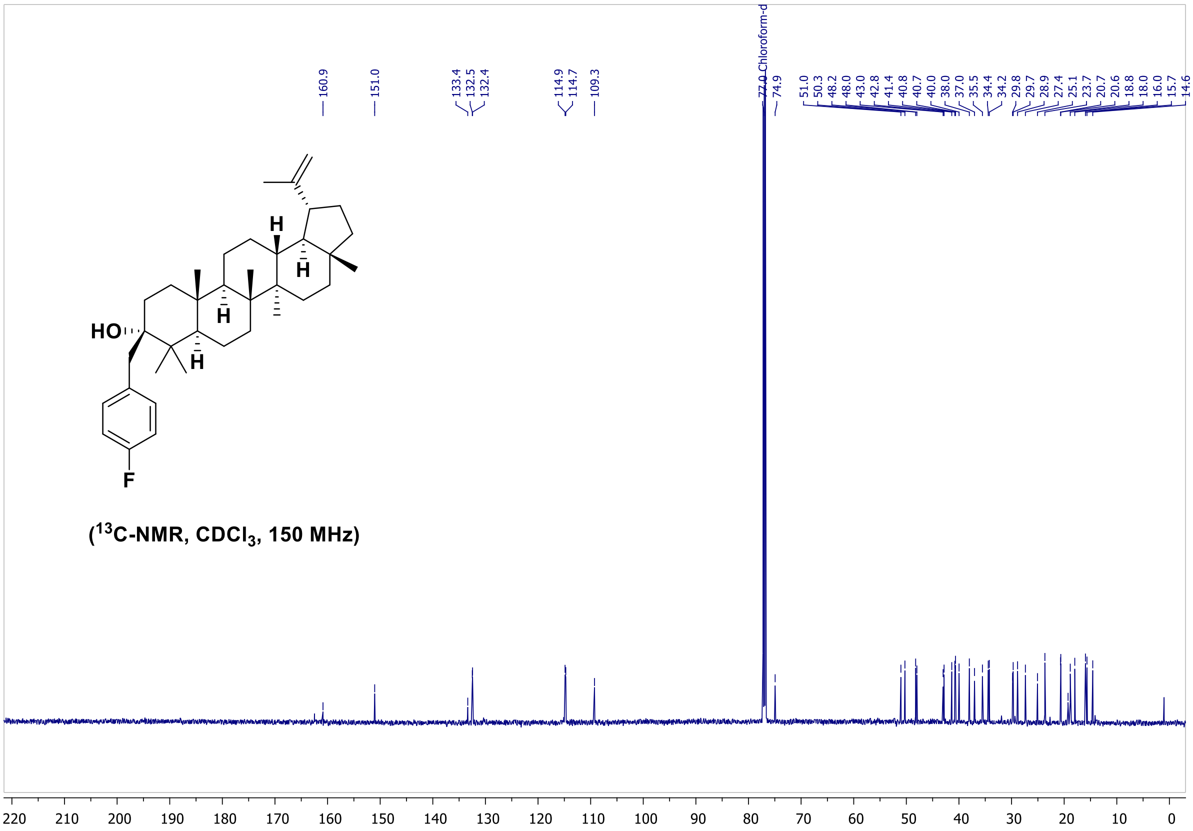

3-allyl lupeol (11)

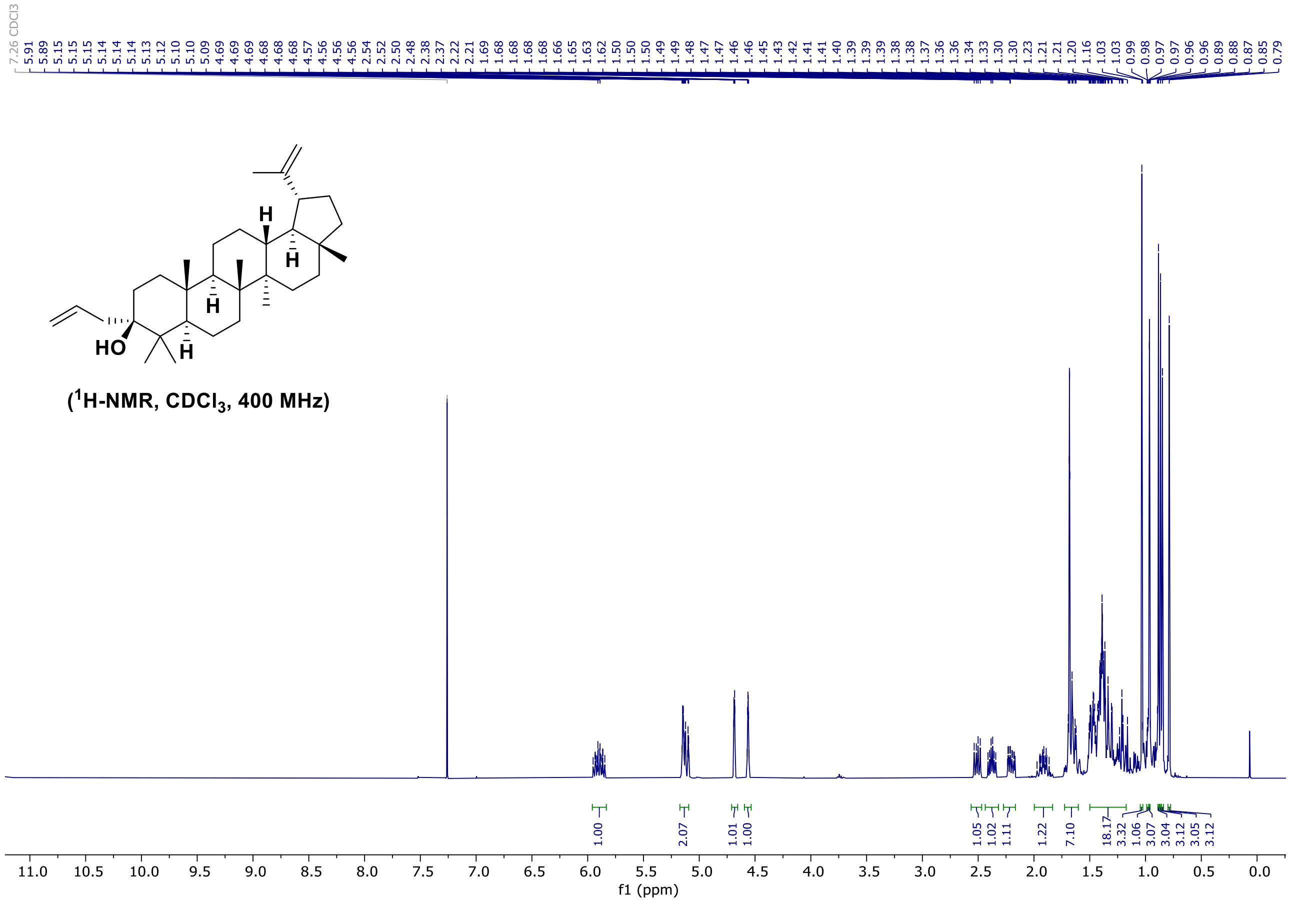

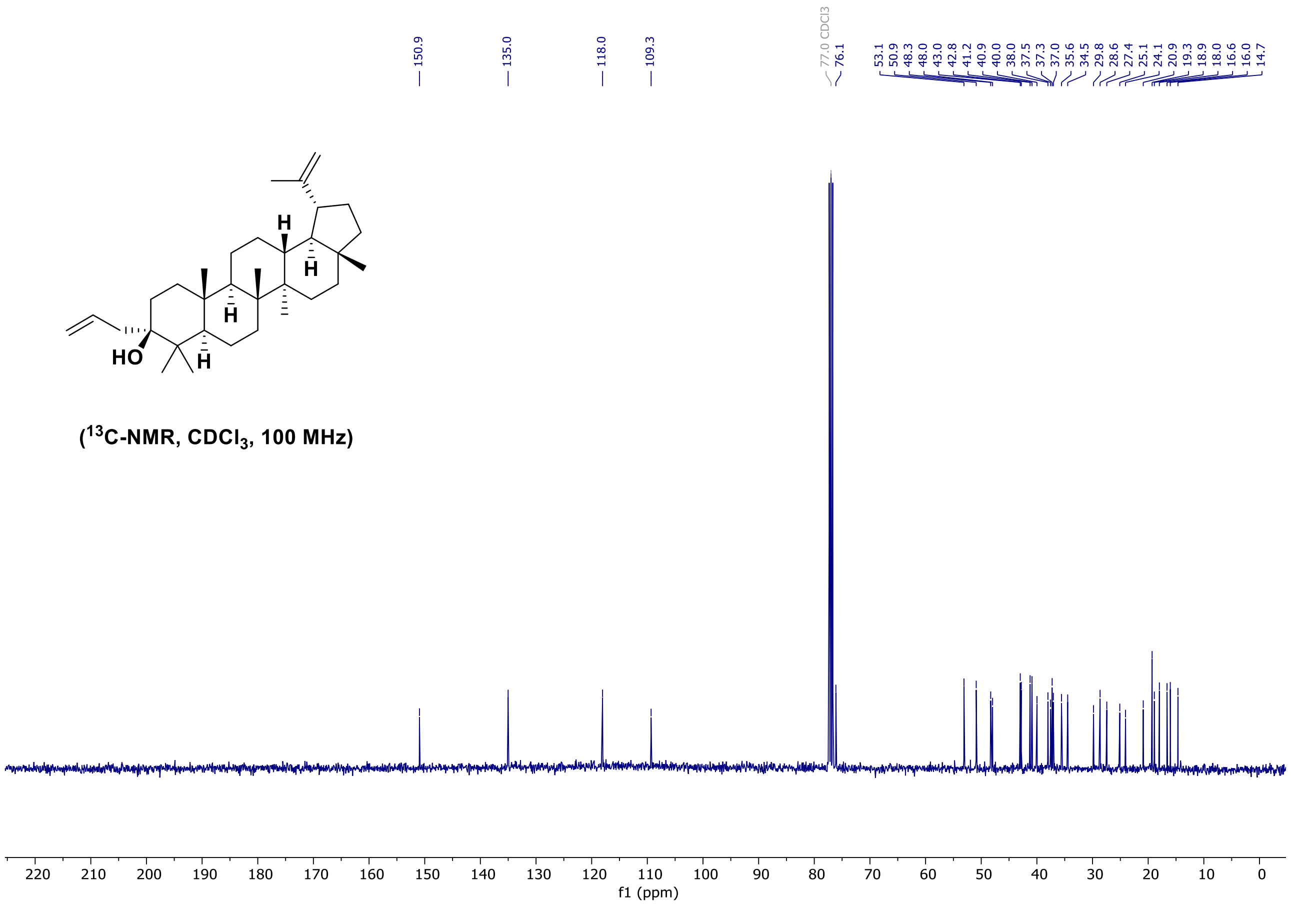

3-epi-3-allyl lupeol (12)

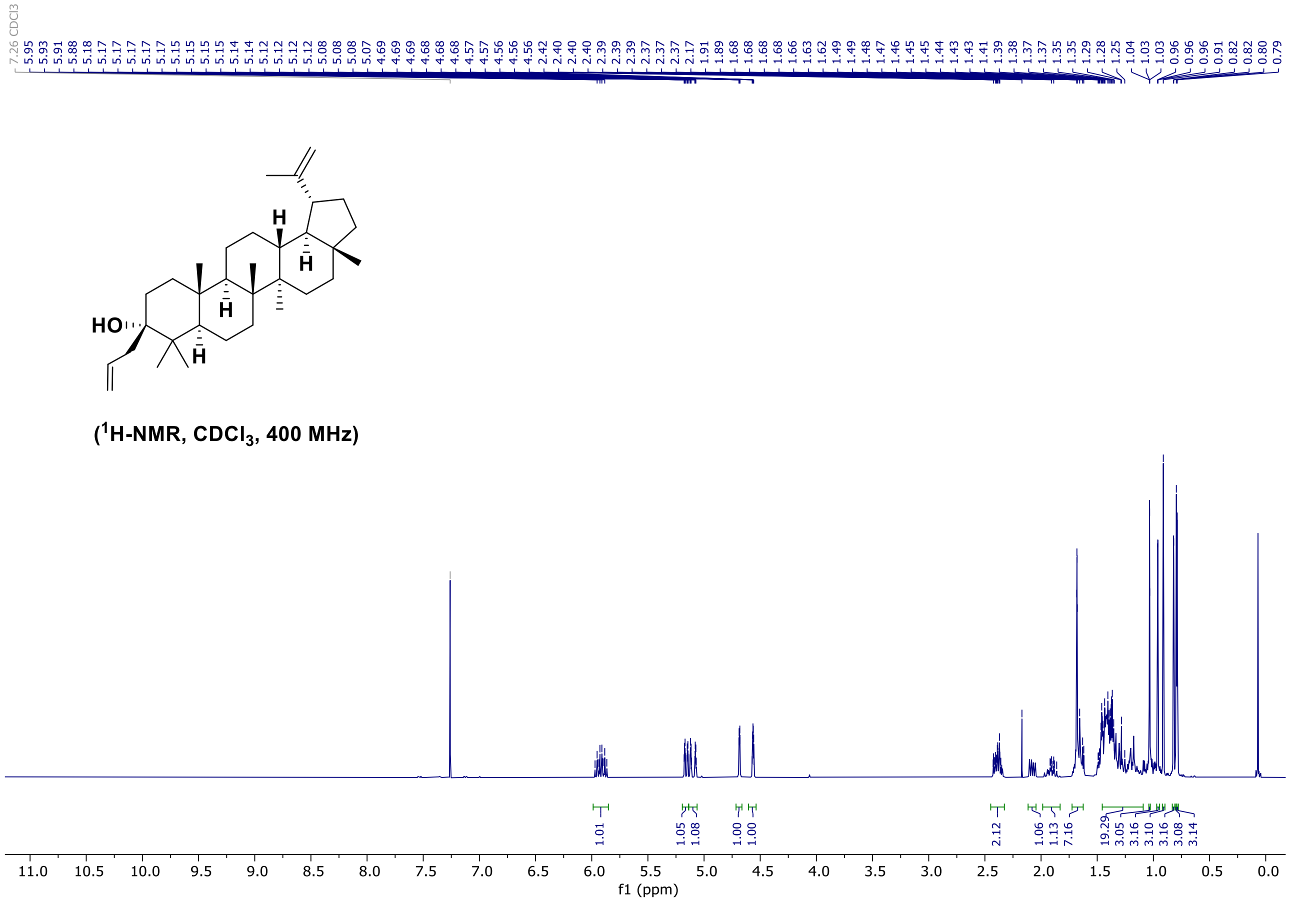

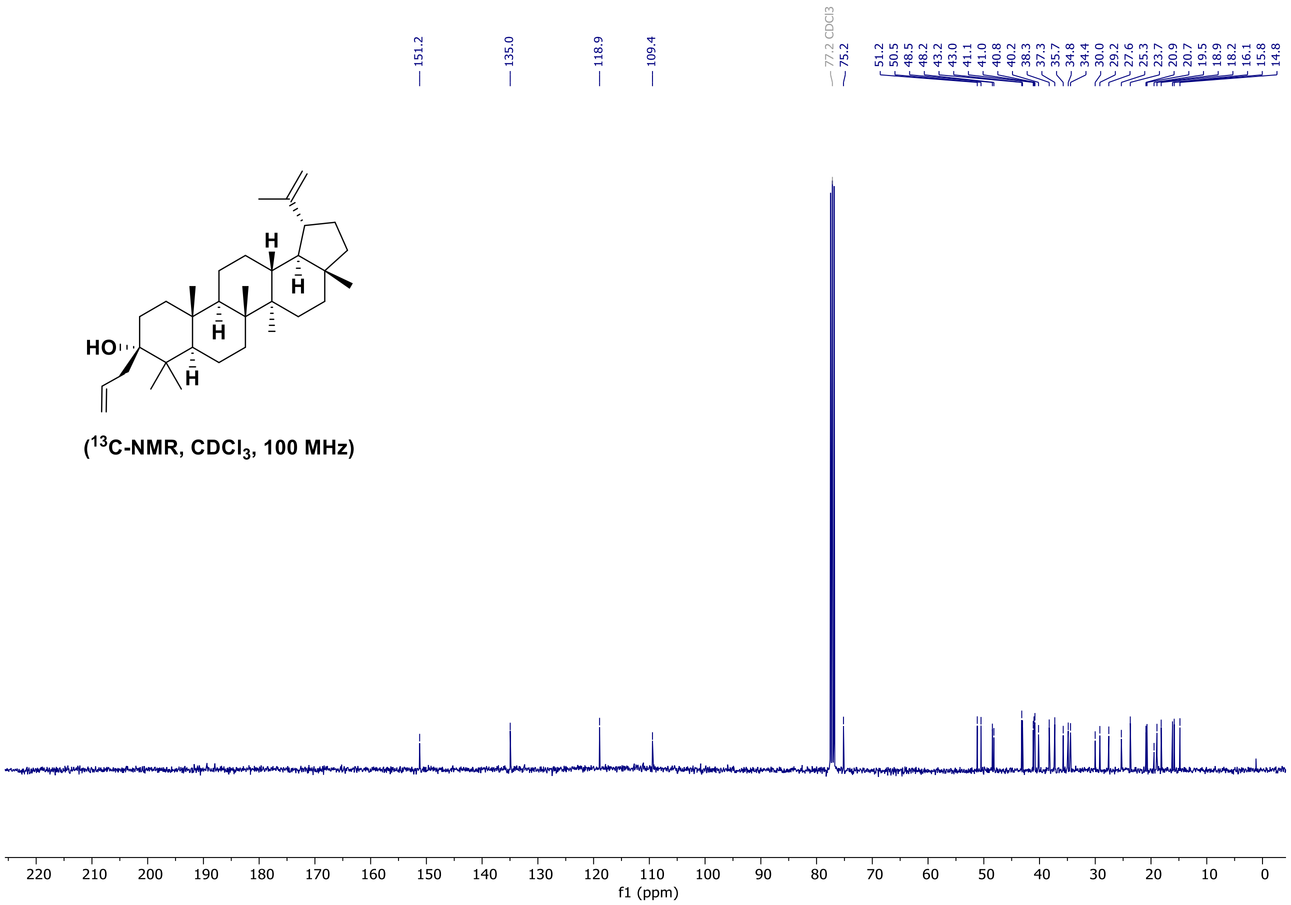

3-(R)-3,1′-epoxy-lup-20-ene (13) and 3-(S)-3,1′-epoxy-lup-20-ene (14)

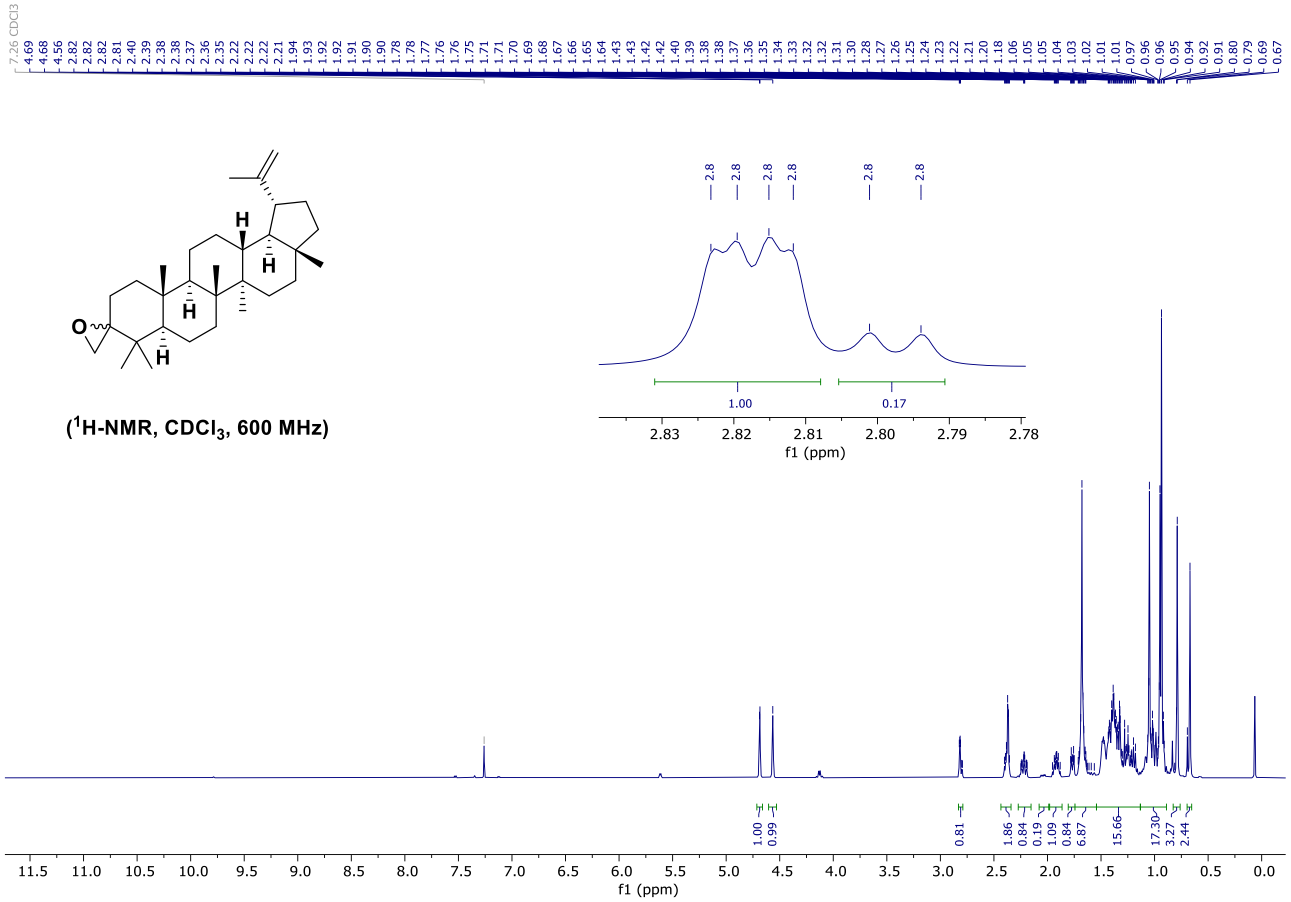

(¹H-NMR, $CDCl_3$, 600 MHz)

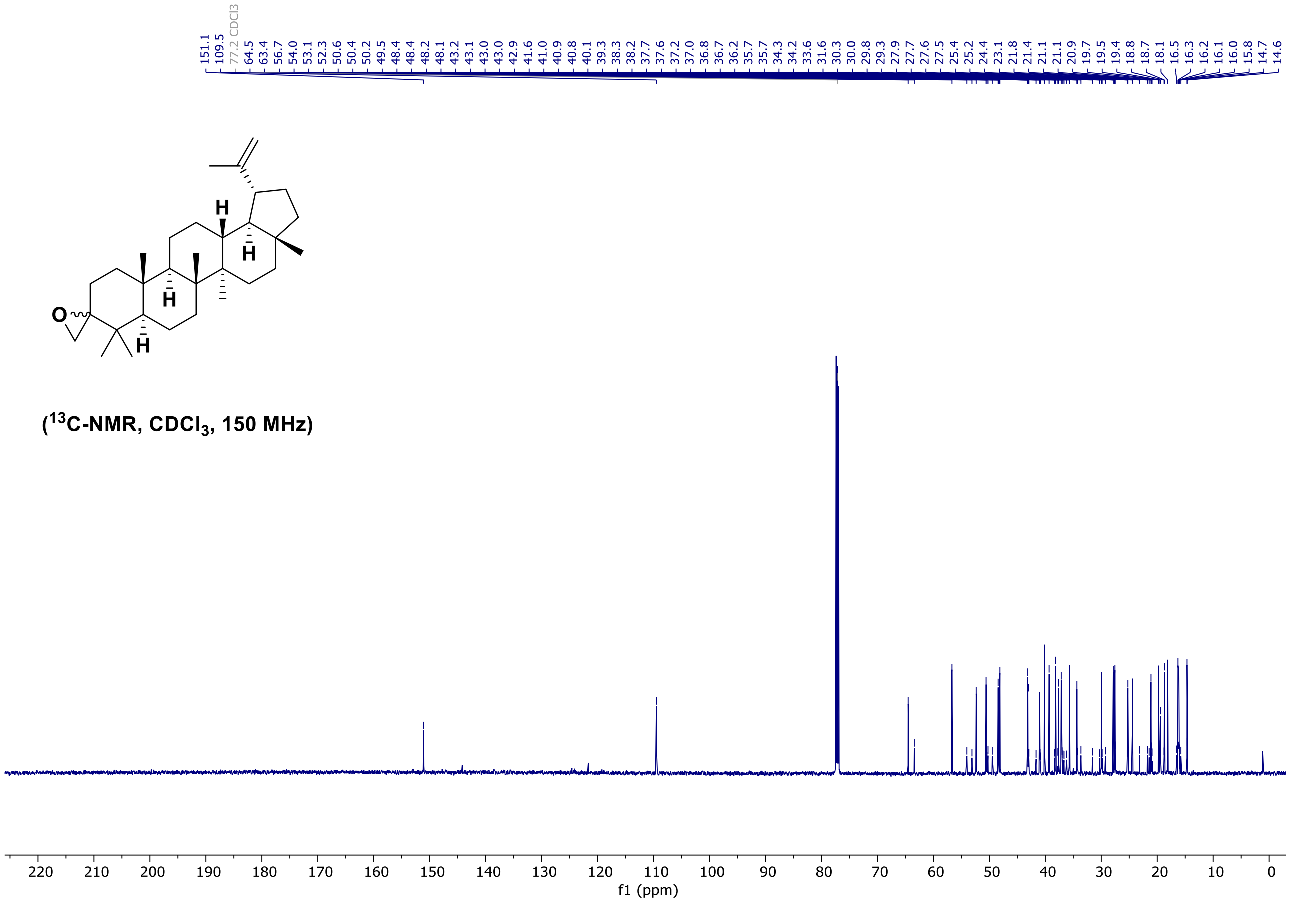

(¹³C-NMR, $CDCl_3$, 150 MHz)

3-hydroxymethyl-lup-1,20-diene (15)

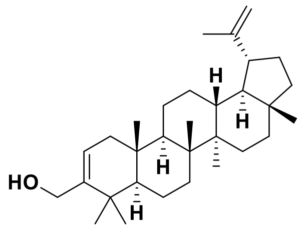

(^{1}H-NMR, $CDCl_3$, 600 MHz)

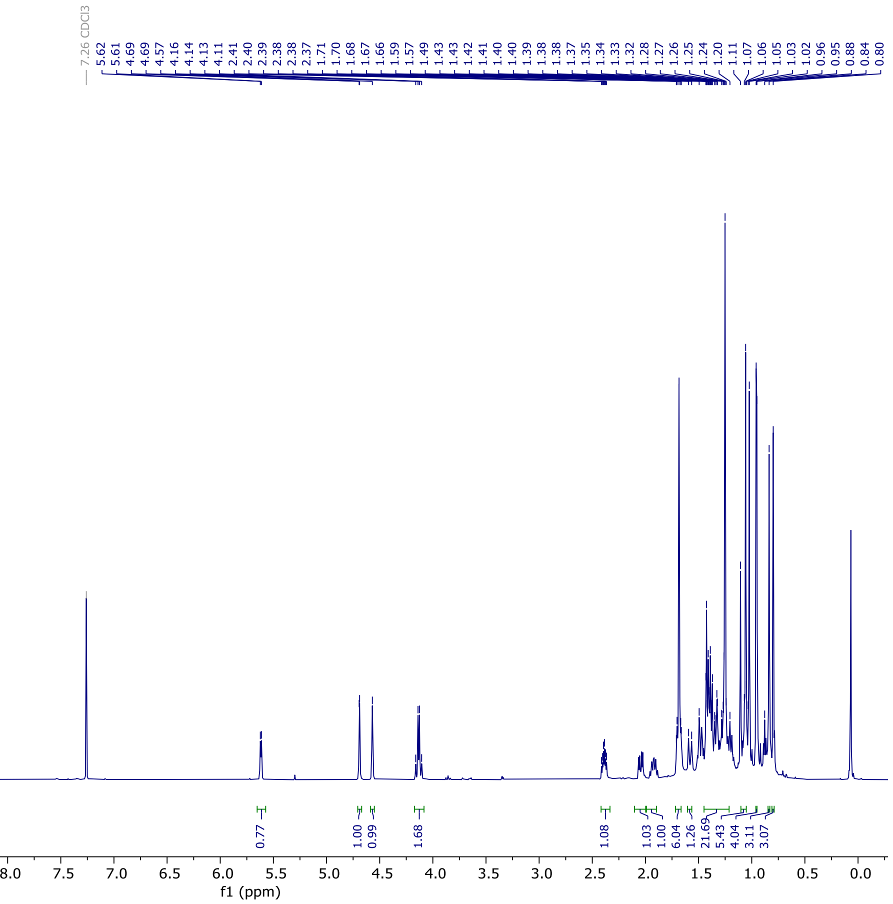

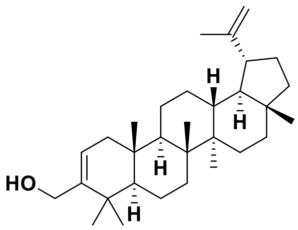

(^{13}C-NMR, $CDCl_3$, 150 MHz)

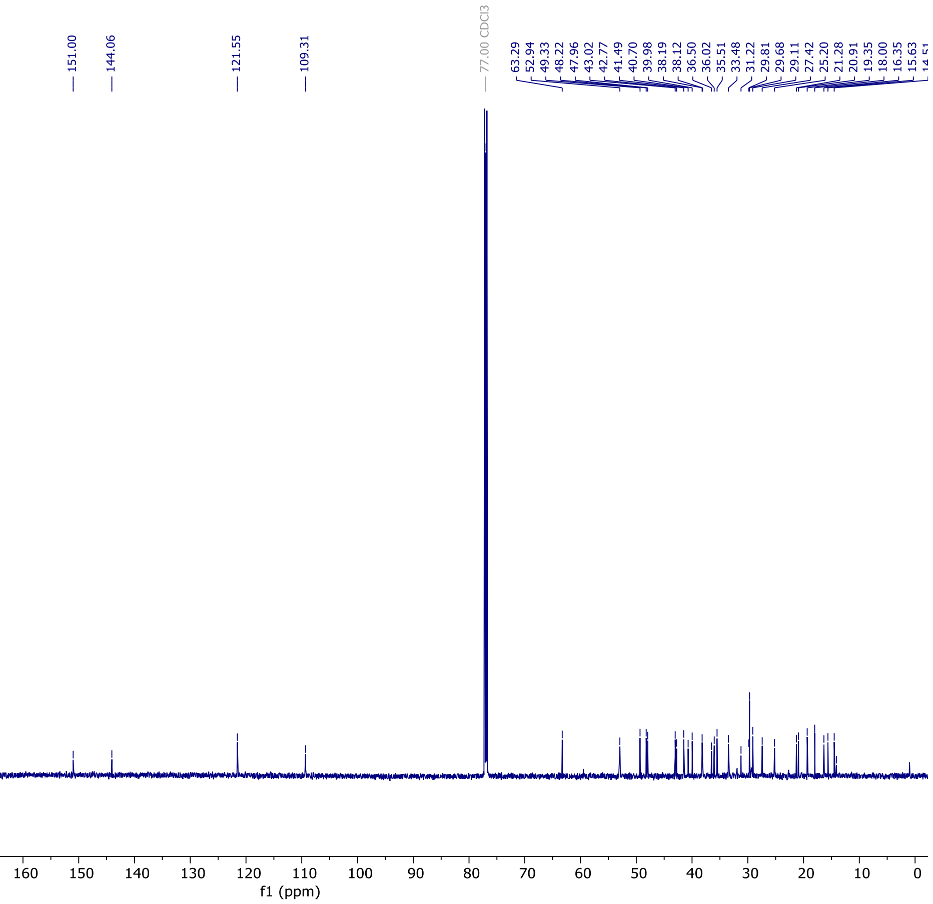

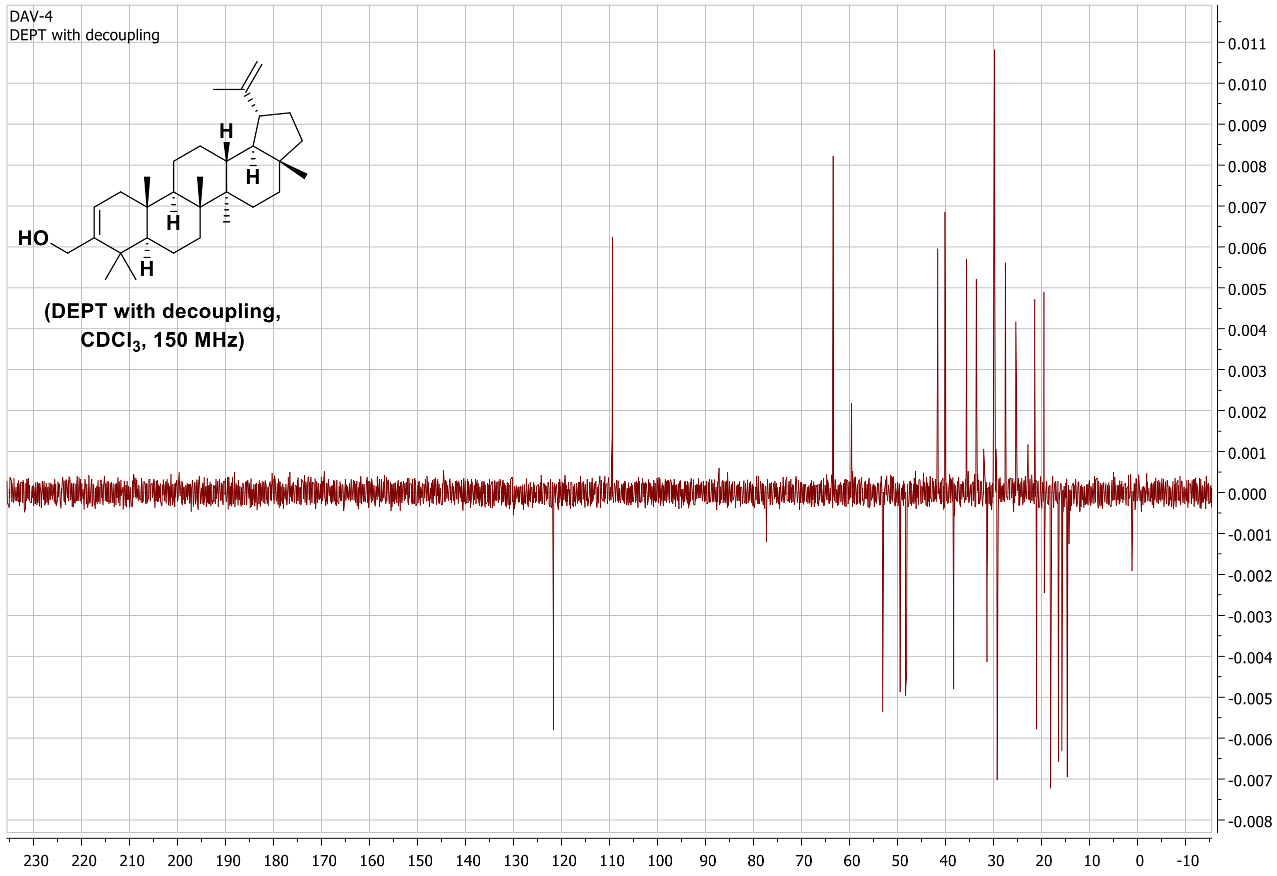
DAV-4
DEPT with decoupling
HO
H
(DEPT with decoupling, CDCl3, 150 MHz)

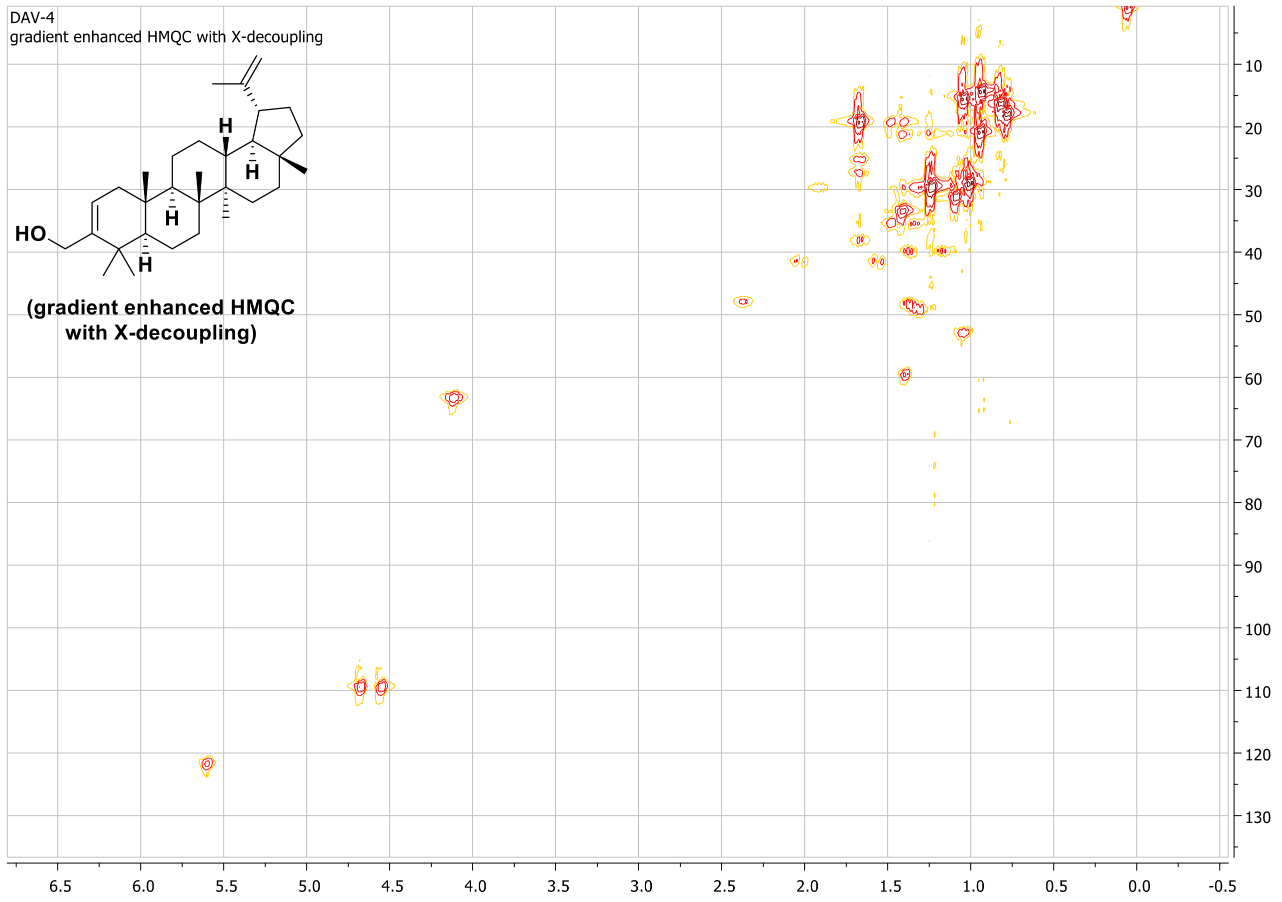
DAV-4
gradient enhanced HMQC with X-decoupling
HO
H
(gradient enhanced HMQC with X-decoupling)

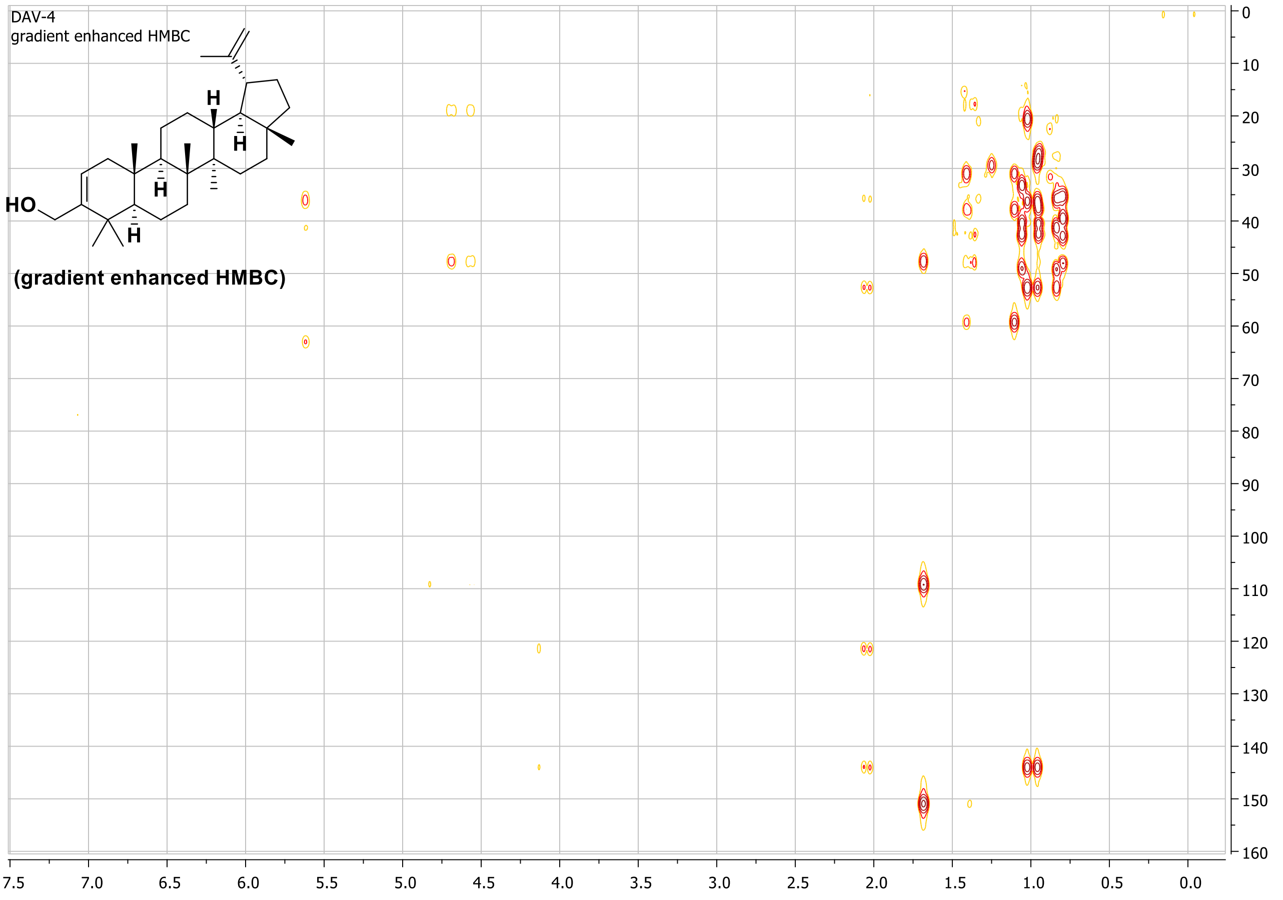
DAV-4
gradient enhanced HMBC
HO
H
(gradient enhanced HMBC)

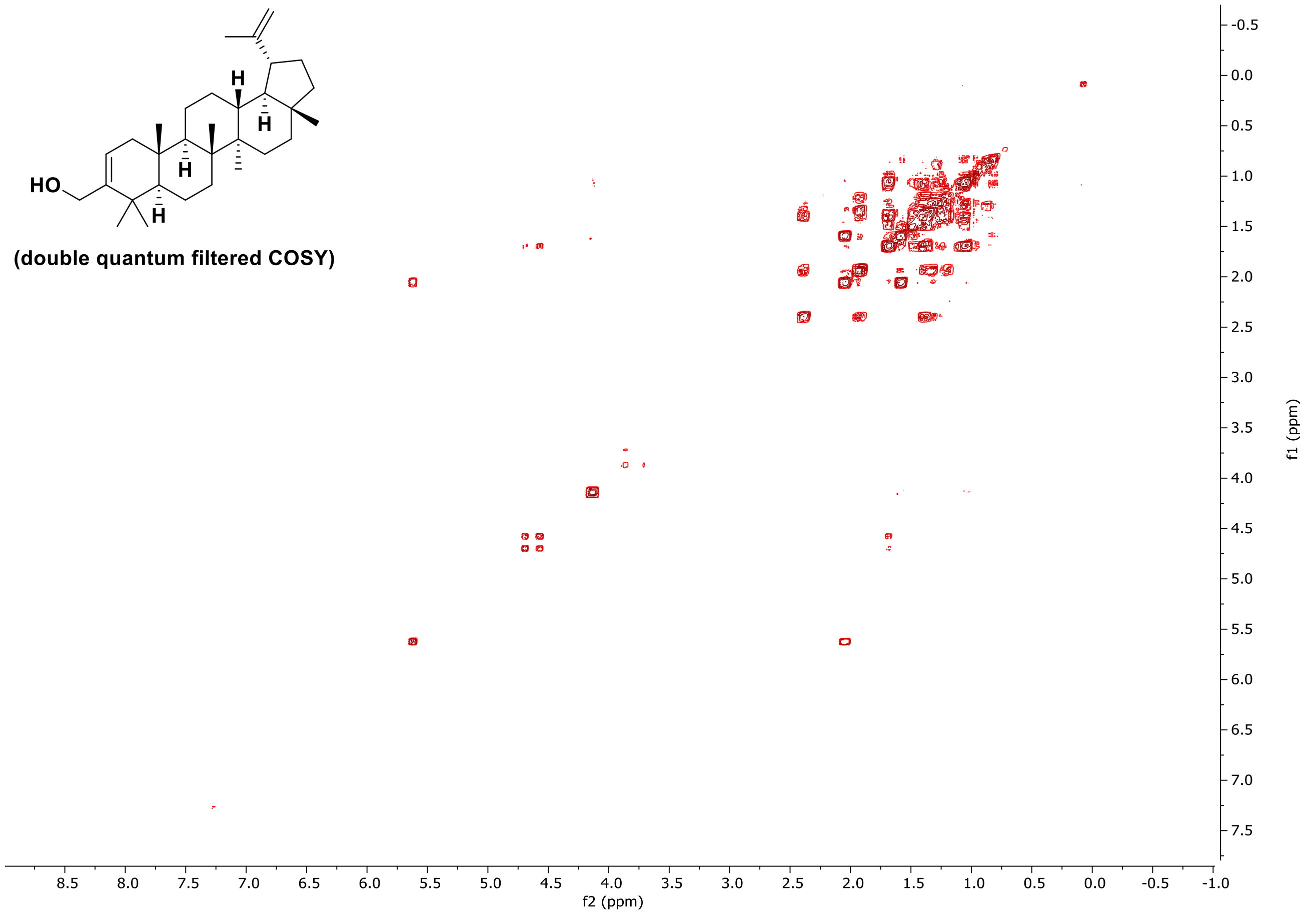
HO
H
(double quantum filtered COSY)
f2 (ppm)
f1 (ppm)

1’,30-epoxy-lupeol (16)

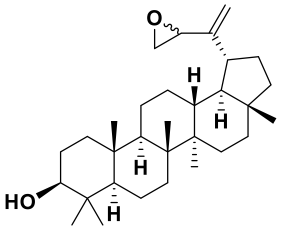

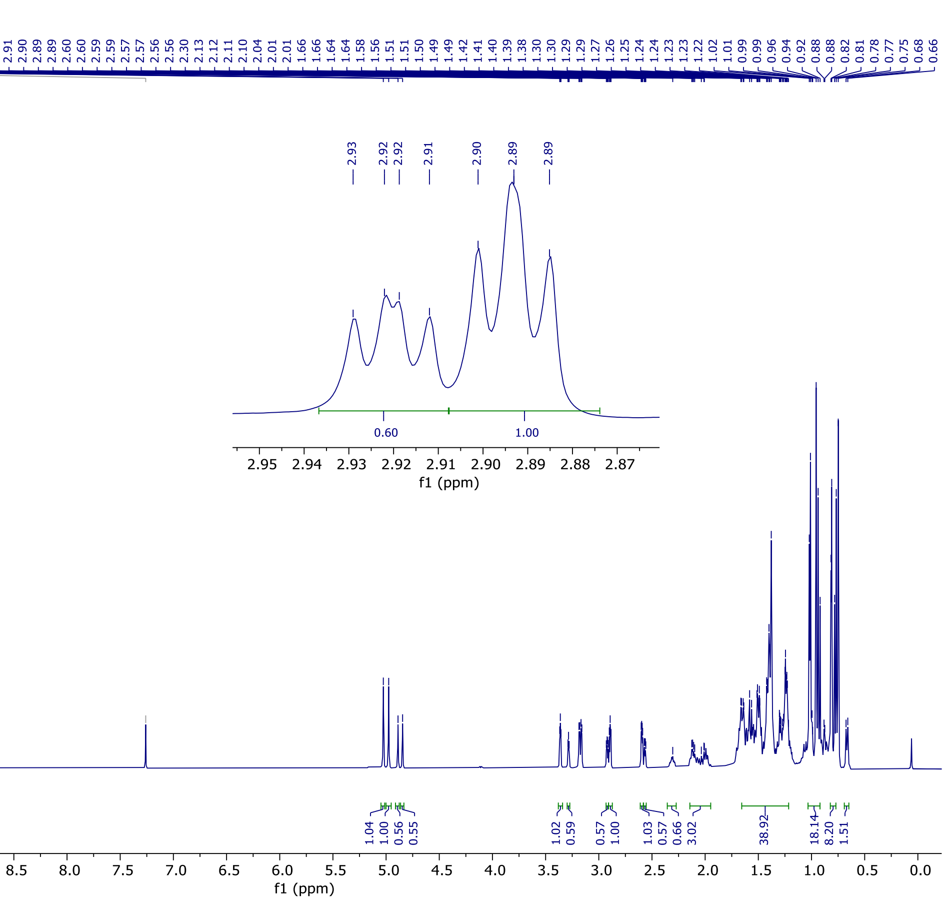

(^{1}H-NMR, CDCl$_3$, 600 MHz)

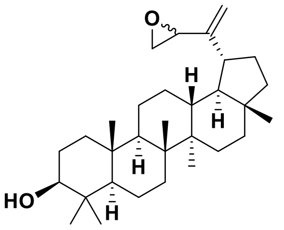

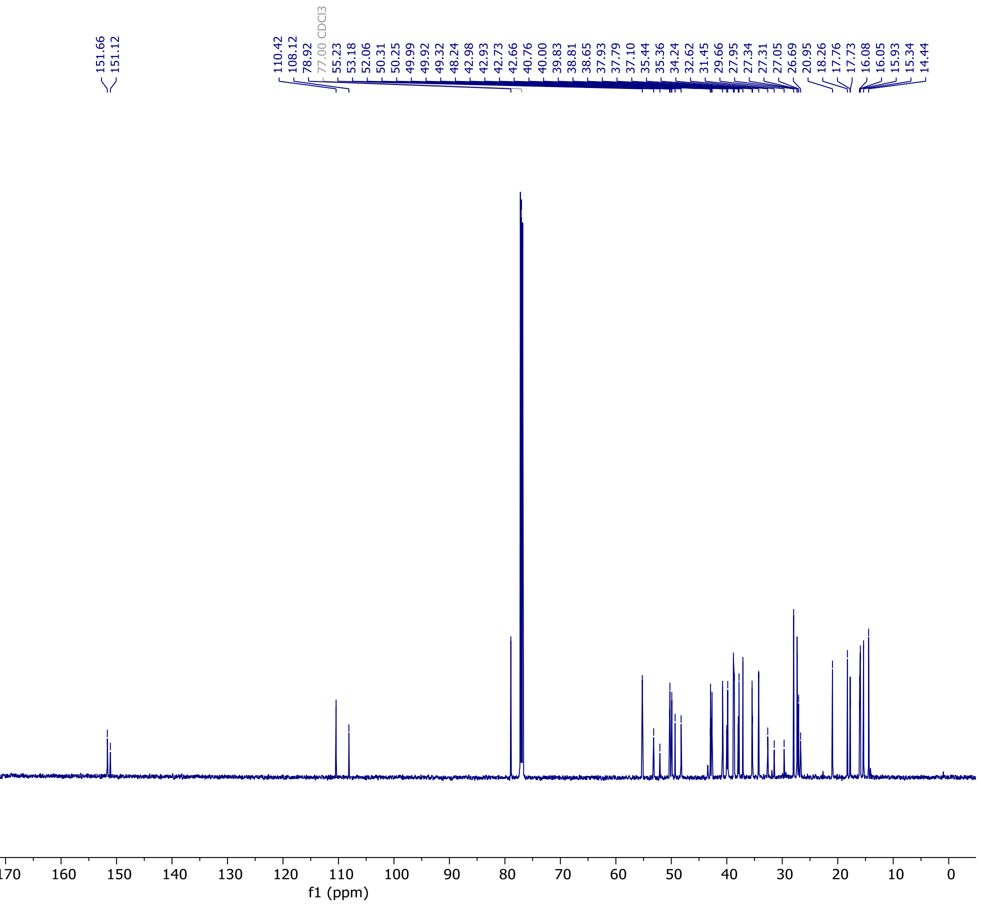

(^{13}C-NMR, CDCl$_3$, 150 MHz)

1’-chloro-30-hydroxy-lupeol (17)

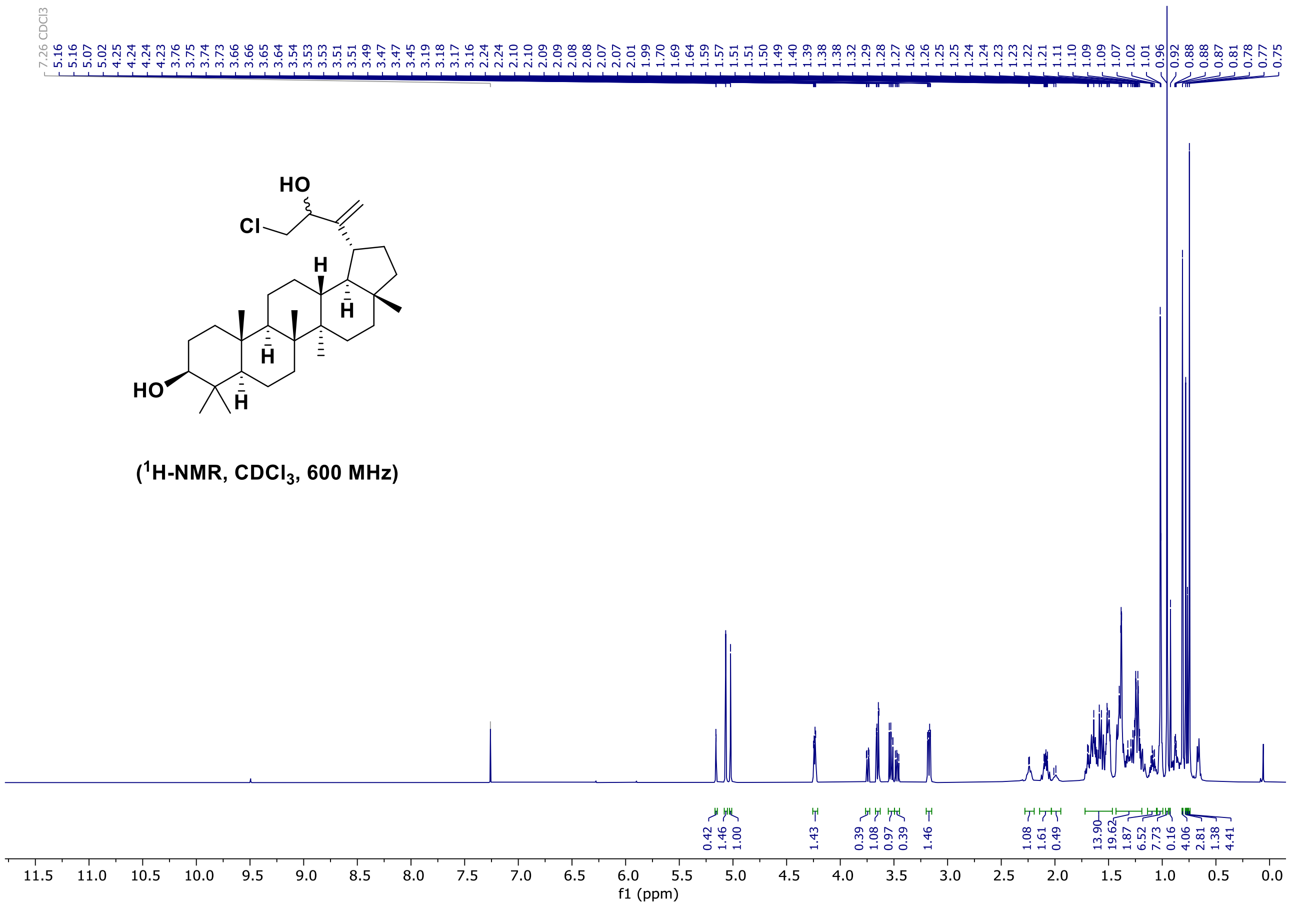

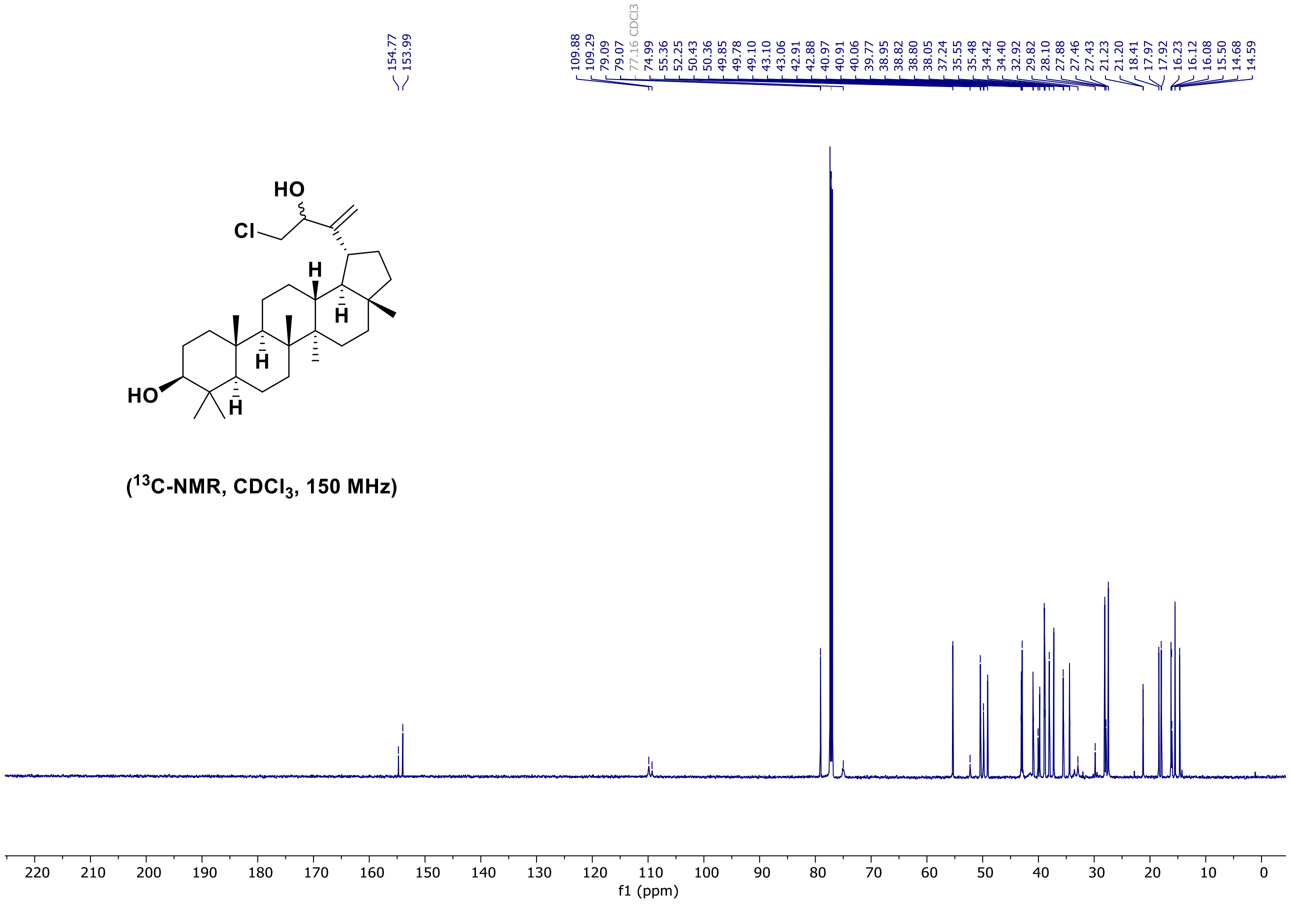

References

(1) (a) Burns, D.; Reynolds, W. F.; Buchanan, G.; Reese, P. B.; Enriquez, R. G. *Magnetic Resonance in Chemistry* **2000**, *38*, 488-493. (b) Surendra, K.; Corey, E. *Journal of the American Chemical Society* **2009**, *131*, 13928-13929.

(2) Khan, M. F.; Mishra, D. P.; Ramakrishna, E.; Rawat, A. K.; Mishra, A.; Srivastava, A. K.; Maurya, R. *Medicinal Chemistry Research* **2014**, *23*, 4156-4166.

(3) Aquilina, J. M.; Smith, M. W. *Journal of the American Chemical Society* **2022**, *144*, 11088-11093.

(4) Wal, A.; Wal, P.; Rai, A. *Journal of Pharmaceutical Sciences and Research* **2010**, *2*, 13.
